# Heparanase inhibition as a systemic approach to protect the endothelial glycocalyx and prevent microvascular complications in diabetes

**DOI:** 10.1186/s12933-024-02133-1

**Published:** 2024-02-01

**Authors:** Monica Gamez, Hesham E. Elhegni, Sarah Fawaz, Kwan Ho Ho, Neill W. Campbell, David A. Copland, Karen L. Onions, Matthew J. Butler, Elizabeth J. Wasson, Michael Crompton, Raina D. Ramnath, Yan Qiu, Yu Yamaguchi, Kenton P. Arkill, David O. Bates, Jeremy E. Turnbull, Olga V. Zubkova, Gavin I. Welsh, Denize Atan, Simon C. Satchell, Rebecca R. Foster

**Affiliations:** 1 Bristol Renal, Bristol Medical School, University of Bristol, Dorothy Hodgkin Building, Whitson Street, Bristol, BS1 3NY United Kingdom; 2 Department of Computer Science, Merchant Venturers Building, University of Bristol, Woodland Road, Bristol, BS8 1UB United Kingdom; 3 Academic Unit of Ophthalmology, Translational Health Sciences, Bristol Medical School, University of Bristol, Biomedical Sciences Building, University Walk, Bristol, BS8 1TD United Kingdom; 4 Sanford Burnham Prebys Medical Discovery Institute, 10901 North Torrey Pines Road, La Jolla, CA 92037 USA; 5 School of Medicine, Biodiscovery Institute, University of Nottingham, Nottingham, NG7 2UH United Kingdom; 6 Centre for Glycoscience, School of Life Sciences, Keele University, Staffordshire, ST5 5BG United Kingdom; 7 Ferrier Research Institute, Victoria University of Wellington, 69 Gracefield Rd, Lower Hutt, 5046 New Zealand; 8 Bristol Eye Hospital, University Hospitals Bristol & Weston NHS Foundation Trust, Bristol, BS1 2LX United Kingdom

## Abstract

**Background:**

Diabetes mellitus is a chronic disease which is detrimental to cardiovascular health, often leading to secondary microvascular complications, with huge global health implications. Therapeutic interventions that can be applied to multiple vascular beds are urgently needed. Diabetic retinopathy (DR) and diabetic kidney disease (DKD) are characterised by early microvascular permeability changes which, if left untreated, lead to visual impairment and renal failure, respectively. The heparan sulphate cleaving enzyme, heparanase, has previously been shown to contribute to diabetic microvascular complications, but the common underlying mechanism which results in microvascular dysfunction in conditions such as DR and DKD has not been determined.

**Methods:**

In this study, two mouse models of heparan sulphate depletion (enzymatic removal and genetic ablation by endothelial specific Exotosin-1 knock down) were utilized to investigate the impact of endothelial cell surface (i.e., endothelial glycocalyx) heparan sulphate loss on microvascular barrier function. Endothelial glycocalyx changes were measured using fluorescence microscopy or transmission electron microscopy. To measure the impact on barrier function, we used sodium fluorescein angiography in the eye and a glomerular albumin permeability assay in the kidney. A type 2 diabetic (T2D, *db/db*) mouse model was used to determine the therapeutic potential of preventing heparan sulphate damage using treatment with a novel heparanase inhibitor, OVZ/HS-1638. Endothelial glycocalyx changes were measured as above, and microvascular barrier function assessed by albumin extravasation in the eye and a glomerular permeability assay in the kidney.

**Results:**

In both models of heparan sulphate depletion, endothelial glycocalyx depth was reduced and retinal solute flux and glomerular albumin permeability was increased. T2D mice treated with OVZ/HS-1638 had improved endothelial glycocalyx measurements compared to vehicle treated T2D mice and were simultaneously protected from microvascular permeability changes associated with DR and DKD.

**Conclusion:**

We demonstrate that endothelial glycocalyx heparan sulphate plays a common mechanistic role in microvascular barrier function in the eye and kidney. Protecting the endothelial glycocalyx damage in diabetes, using the novel heparanase inhibitor OVZ/HS-1638, effectively prevents microvascular permeability changes associated with DR and DKD, demonstrating a novel systemic approach to address diabetic microvascular complications.

**Supplementary Information:**

The online version contains supplementary material available at 10.1186/s12933-024-02133-1.

## Background

Currently, over 8% of the global adult population live with diabetes mellitus [1]. Diabetes negatively impacts cardiovascular health and as a result, is a risk factor for the development of microvascular diseases which impact multiple vascular beds. Two of the most common microvascular complications of diabetes are diabetic retinopathy (DR) and diabetic kidney disease (DKD). DR is the leading cause of vision impairment and vision loss in the working age population, and affects over 34% (approximately 93 million people) of the diabetic population globally [2]. DKD impacts up to 40% of diabetic patients (> 116 million people), and is the leading cause of end stage renal failure [3].

Both DR and DKD are characterized by microvascular barrier dysfunction, resulting in increased vascular permeability. The blood-retina-barrier is formed of pericytes which wrap around a basement membrane, and a monolayer of non-fenestrated endothelial cells with junctions, forming a relatively impermeable barrier. In DR, a breakdown in this barrier results in abnormal vascular leak of fluid and circulating proteins, leading to diabetic macular oedema (DME) [4]. In the kidney, the glomerular filtration barrier is composed of specialised epithelial cells called podocytes with interdigitating podocyte foot processes, which wrap around the glomerular basement membrane and fenestrated endothelial cells. It is highly permeable to water and small solutes but not to macromolecules. DKD is characterised by increased urinary albumin excretion (albuminuria), resulting from damage to the glomerular filtration barrier causing increased permeability [5]. Prospective and epidemiological studies in people with diabetes have shown that development of microalbuminuria, *i.e.* small quantities of albumin in the urine, is an independent predictor of progressive renal disease [6, 7].

As both DR and DKD have microvascular barrier complications, it is perhaps unsurprising that the incidence of one complication significantly increases the likelihood of developing the other [8–10]. Studies suggest DR and DKD have common mechanisms in their pathogenesis, as improved glycaemic and blood pressure control reduces the incidence of both [11, 12]. There are no preventive treatments for DR or DKD. Retinal laser therapy and anti-VEGFA treatments remain the most effective treatments for sight-threatening DR [13, 14], as they reduce DME, the most common cause of blindness in DR [14]. However, laser therapy is inherently destructive and 40–50% of patients with DME do not fully respond to anti-VEGFA treatments [4]. The incidence of DR increases with duration of diabetes, with a prevalence of 80% in patients who have had diabetes for > 15 years [15], suggesting that early intervention may reduce DR development. Similarly, development of DKD usually occurs more than 10 years after the onset of diabetes, providing an opportunity for early intervention. Current DKD treatments such as sodium-glucose cotransporter-2 inhibitors (SGLT2i), renin-angiotensin system (RAS) inhibitors, and mineralocorticoid receptor antagonists (MRA) have renoprotective effects beyond those attributable to blood pressure lowering alone [16]. However, despite available treatments, DKD still progresses towards kidney failure in a proportion of patients. As both DR and DKD involve microvascular barrier dysfunction, there is potential for identification of therapeutic targets which are suitable for systemic prevention/treatment of diabetes associated microvascular barrier dysfunction in general. As glomerular and retinal vessel architecture is distinct from one another, these vessel beds allow for the opportunity to explore common mechanisms of widespread microvascular dysfunction.

The endothelial glycocalyx (eGlx) lines the luminal surface of all vascular endothelial cells. It has emerged as a key determinant of endothelial and vascular health, including through its role in barrier function [17, 18]. The eGlx is a multifunctional structure serving as; a protective layer on endothelial cells, a regulator of immune cell and molecule adhesion, as well as a barrier to macromolecules in circulating blood [17, 19, 20]. The eGlx limits endothelial cell activation by cytokines, and damage to this protective layer has been linked to endothelial dysfunction and increased permeability [21, 22], a key characteristic of both DR and DKD. The eGlx is a carbohydrate rich layer comprised primarily proteoglycans and glycosaminoglycans (GAGs) including heparan sulphate (HS), chondroitin sulphate, and hyaluronic acid [18]. HS chain elongation is a key step in HS synthesis and is carried out by HS-polymerase, a heterodimer made up of two glycosyltransferases, Exotosin-1 (Ext1) and Exotosin-2 (Ext2) [23]. Upon completion of HS synthesis in the Golgi apparatus, membrane HS proteoglycans are transported to the cell surface.

Although the matrix metalloprotease (MMP) enzyme family can cleave HS proteoglycan core proteins found in the eGlx such as Syndecan-4 [24, 25], heparanase-1 is the only known mammalian endoglycosidase with the capability to directly cleave HS, and is systemically upregulated in diabetes. Heparanase is elevated in the vitreous and epiretinal membranes of DR patients as well as the kidneys of DKD patients [26–28]. We hypothesised that eGlx HS plays a key role in the barrier properties of retinal and glomerular capillaries and that inhibiting heparanase to protect eGlx HS, could serve as a novel systemic approach to protect against microvascular complications in diabetes.

Here, we show that HS plays a key role in microvascular barrier function of the eGlx in vivo*.* We demonstrate for the first time that the eGlx can be therapeutically targeted with the novel heparanase inhibitor OVZ/HS-1638, a unique tetravalent dendrimer heparanase inhibitor with no-off target anticoagulant activity [29], to prevent microvascular dysfunction in diabetes in multiple vessel beds, *i.e.* eye and kidney, in a mouse model of type-2 diabetes.

## Research design and methods

### Animals

Animals were kept according to the *Guidelines on the Use of Animals in Research* and studies performed in compliance with the University of Bristol’s guidelines and the Animal (Scientific Procedure) Act 1986 under licences approved by UK Home Office.

C57BL/6J and FVB mice (mean weight 25 g) were obtained from Charles Rivers (Charles Rivers Laboratories, Wilmington, MA). BKS.Cg-+*Lepr*^*db*^*/*+*Lepr*^*db*^/OlaHsd (*db/db*) and lean *db/* + littermate controls (lean) were obtained from Envigo (Huntindon, England). Endothelial specific Ext1 conditional knockout mice (*Ext1*^*(ECKO*)^) and control mice were generated on a C57BL/6 background by crossing *Tie2*-Tet-O-*Cre* mice with *Ext1*-floxed (*Ext1*^*fl/fl*^) [30] mice and bred in house by the Animal Service Unit at the University of Bristol.

### Heparinase III enzyme treatments

Six-week-old FVB mice were used for transmission electron microscopy (TEM) kidney studies and C57BL/6J mice were used for fluorescein angiography experiments due to incompatibility of albino FVB mice with fluorescein angiography analysis. Mice were given retro orbital injections (under anaesthesia) of heparinase III derived from Flavobacterium (H8891, Sigma-Aldrich, St. Louis, MO) reconstituted in PBS at 8.2 units/kg or heat-inactivated enzyme as negative control. After 30-min, mice were prepared for cardiac perfusions or sodium fluorescein angiography as described below.

### Targeted knockout of Exotosin-1 in endothelial cells

*Tie2-rtTa*^+/-^*, Tet-Cre*^+/-^*, Ext1*^*fl/fl*^ (*Ext1*^*(ECKO*)^) and age/sex-matched littermate controls (LMC) which lacked at least one transgene, were given 2 mg/ml of doxycycline with 5% sucrose in drinking water for three weeks to induce knockout.

### Urine collection and urine albumin creatinine ratio

Urine was collected and urine albumin creatinine ratios (uACR) were quantified as previously described [31]. Briefly, albumin concentration was measured using Mouse Albumin ELISA kit (E90-134, Bethyl Laboratories, Inc) and creatinine concentration measured at Langford Vets Diagnostic Laboratories (The University of Bristol). Two urine samples in the diabetes study (one *db/db* sample and one *db/db* + HI sampled) were invalidated due to bacterial contamination and therefore omitted.

### l-lysine treatments

l-lysine was injected via intraperitoneal (i.p.) route, with 2 g/kg of sterile _L_-lysine (L5751, Sigma-Aldrich) dissolved in PBS, a dose previously shown to be effective at inhibiting tubular reabsorption in mice [32]. ‘Post-treatment’ urine was then collected after _L_-lysine treatment over the course of 2-h in metabolic cages.

### Blood glucose measurements

Weekly blood glucose measurements were taken using Accu-Check standard glucometer (range 0–40 mmol/L) with Accu-Check AVIVA test strips (Roche Diagnostics Limited, West Sussex,England).

### OVZ/HS-1638 heparanase inhibitor treatments

Seven-week-old BKS.Cg- + *Lepr*^*db*^/ + *Lepr*^*db*^/OlaHsd (*db/db*) (n = 10, mean weight 45 g) were randomly assigned to experimental groups (vehicle or OVZ/HS-1638) and caged accordingly. Heparanase inhibitor OVZ/HS-1638 (Additional file 1: Fig. S2, described previously as compound 29) [29] was reconstituted in PBS at 20 mg/kg and administered to nine-week-old *db/db* mice (n = 5) for 2 weeks via i.p. injection. *Db/db* vehicle control mice (n = 5) and non-diabetic lean mice (n = 5) were administered PBS only.

### Cardiac perfusions for eGlx staining and transmission electron microscopy

Mice under anaesthesia were cardiac perfsued with Ringer’s solution to remove red blood cells from the circulation, followed by Alcian blue in fixative solution (0.15 M sodium cacodylate pH 7.4, 2.5% glutaraldehyde, 0.01 g/mL of Alcian Blue 8GX (75,881–23-1, Santa Cruz Biotechnology, Dallas, TX) in water) to stain the eGlx in vivo, as previously described [31]. Mouse retina and kidney samples were removed, and tissue was processed for TEM at the University of Bristol Wolfson Bioimaging Facility. See Additional file 1: Methods S1 ‘Processing of samples for transmission electron microscopy’ for full details.

Researchers were blinded for imaging and analysis. High power images (49,000 × magnification) were acquired on a FEI Tecnai-12 BioTwin Spirit transmission electron microscope (ThermoFisher Scientific, Hillsboro, OR). To standardise eGlx depth measurements, images were overlayed with a grid on ImageJ, and measurements taken where intersecting grid lines also intersected lipid bilayers (Additional file 1: Fig. S1). See Additional file 1: Method S1 ‘Electron microscopic glomerular filtration barrier measurements’ for more details.

### Immunofluorescence for HS on mouse kidney and retinal tissue

Mouse kidney tissue was fixed in 4% formaldehyde (PFA), paraffin embedded, and cut into 3 μm sections. Slides were deparaffinized in Xylenes and rehydrated. A panel of antibodies was initially tested to determine which antibody bound to the eGlx HS (data not shown). The vesicular stomatitis virus G (VSV-G) tagged phage display antibody HS3A8V derived from bovine kidney HS [33, 34] (gift from Jerry Turnbull, Keele University) detected HS in the eGlx in this model. HS3A8V was diluted 1:5 and incubated at 4 °C overnight. All washes were with PBS, pH 7.2. Secondary rabbit anti-VSVG (A190-131A, Bethyl Laboratories, Inc., Montgomery, TX) was incubated for 1 h at 1:50 at room temperature, washed, and tertiary anti-rabbit 488 (A-11001, ThermoFisher Scientific, Waltham, MA) incubated for 1 h at 1:250. Tissue was counterstained in 300 nM 4’,6-Diamidino-2-Phenylindole, Dihydrochloride (DAPI) (D1306, ThermoFisher Scientific) for 5 min followed by a 15-min membrane stain with Octadecyl Rhodamine B Chloride (R18) (O246, ThermoFisher Scientific) diluted 1:1000 in PBS. Slides were washed and mounted with Pro long gold (P10144, ThermoFisher Scientific) and imaged on a Multi-Laser CLSM Leica SP5 confocal microscope (Leica Microsystems, Wetzlar, Germany).

Mouse eyes were enucleated and retinas isolated. Retinas were flat mounted and fixed in 4% PFA and stained following the same procedure as above, with the VSV-G tagged phage display antibody RB4Ea12V derived from mouse skeletal muscle (gift from Jerry Turnbull, Keele University). A different HS antibody was used in the retina than in the kidney due to the wide variation of HS epitopes which vary between different tissue structures, as has been previously demonstrated [35]. Retinas were coverslip mounted with Pro long gold (P10144, Thermo-Fisher) and imaged on a Carl Zeiss 700 confocal microscope provided by the Kansas State University Confocal Core.

### Lectin staining for eGlx measurements in mouse retinas

Retinas were fixed in 4% PFA, paraffin embedded, and sectioned at 3 µm or flat mounted. Sections were deparaffinised in Xylenes and rehydrated and blocked in 1% BSA. We used FITC-conjugated Lycopersicon esculentum lectin (LEL) (L0401, Merk Life Science, Dorset, UK) to measure changes in eGlx depth. LEL was incubated at 1:200 in 1% BSA overnight and washed in 0.1% Tween-20 PBS. Sections were counterstained with DAPI as above, followed by a 15-min cell membrane stain with R18 (ThermoFisher Scientific) diluted 1:1000 in PBS. Slides were washed in PBS and coverslip mounted. Retinal flat mount (Additional file 1: Fig. S3) was stained in a similar manner excluding R18, and coverslip mounted.

Researchers were blinded for imaging and analysis. Glycocalyx depth was measured as previously described [25, 36, 37]. Briefly, confocal images were taken using an 100X oil immersion objective on a Multi-Laser CLSM Leica SP5 (Leica Microsystems). Using ImageJ, fluorescence peak measurements were obtained using the line tool to draw a line perpendicular to the vessel wall. Fluorescence profiles were plotted for both green filter (LEL staining) and red filter (R18 staining) images (Fig. 2d). EGlx staining was evident by staining on luminal surface of endothelial cells. The distance between the two fluorescence peaks was measured (Fig. 2d), providing a representation of eGlx depth [37]. A minimum of five vessels per animals were analysed. EGlx depth measurements were averaged per mouse.

### Fluorescein angiography for solute flux measurements

A Micron IV imaging system (Phoenix Research Labs, Pleasanton, CA) was used for fluorescein angiography. The following method of imaging was adapted from Allen et al*.* [38]. Briefly, 30-min prior to imaging, mouse pupils were dilated with Tropicamide 1%w/v (Bausch and Lomb, Rochester, NY). Mice were anesthetized and positioned on a stage for imaging. The centre of the image was focused on the optic nerve of the left eye using the brightfield fundus setting. Images were scanned for abnormalities and optical coherence tomography images were taken. The camera was then switched to the green filter and mice were given 50 µl of 0.22 μm sterile filtered 10% Sodium Fluorescein diluted in water by i.p. injection. Recordings at 15 frames per second commenced immediately until the fundus image was saturated with fluorescein (approximately 4- minutes). All camera settings remained constant for the duration of the experiment.

Researchers were blinded for video analysis. The ratio of fluorescence intensity measured in tissue next to the main vessel (V_2_) and within the main vessel (V_1_) (Fig. 2f) was plotted against time to give the change in fluorescence intensity over time (∆V_2/1_/∆t) = (∆V_R_/∆t). To minimize the effect of differential vessel filling time from animal to animal, which may impact solute flux calculations, only data points after the main vessel had reached a steady state were used (*i.e.* when the curve began to plateau but before saturation). This exact point was defined as the point at which the R^2^ of the curve was closest to 1. Similar to Allen et al. [38], we called this time = 0. Solute flux was then calculated using ∆V_R_/∆t from the slope of the linear change in fluorescence intensity during a 200-s window after t = 0. We developed an automated program for this analysis to allow for a larger area of the retina to be analysed and a copy of this program is available upon request.

### Ex vivo glomerular albumin permeability assay

The following assay was previously described in depth by Desideri et al*.* [39]. In brief, mice were anaesthetised and cardiac perfused with Ringer’s solution as above. Kidneys were placed into cold Ringer’s solution with 4% BSA, pH 7.4. Glomeruli were isolated by graded sieving and resuspended in 4% BSA ringer containing 36.5 µg/mL R18 (ThermoFisher Scientific) for 15 min at 4 ºC, to label cell membranes. Glomeruli were washed with 4% BSA ringer and incubated in 4% BSA ringer containing 30 µg/mL AlexaFluor 488 BSA (A13100, ThermoFisher Scientific) for 15 min at 4 ºC to allow fluorescently labelled BSA to diffuse into capillaries. Researchers were blinded for analysis. Albumin permeability across single glomerular capillaries was measured as previously described [39]. Briefly, an individual glomerulus was trapped using a glass capillary pipette on a thin glass bottom petri dish and washed with unlabelled 4% BSA. Videos recorded on Nikon Ti-E inverted confocal microscope (Nikon instruments Inc., Melville, NY). The rate of decline of fluorescence intensity in individual capillary loops was used to calculate apparent glomerular albumin permeability.

### Albumin staining and analysis for retinal extravascular albumin measurements.

Mouse retina sections were deparaffinised and rehydrated as described above. Tissue was permeabilised and blocked with 0.3% Triton-X, 3% BSA in PBS solution for 30-min. Goat anti-mouse albumin (A90-134A-17, Bethyl Laboratories, Inc., Montgomery, TX) was incubated at 1:200 in blocking buffer overnight at 4 °C in a humidity chamber. Sections were washed in 0.3% Triton-X in PBS. Donkey anti-goat Alexa Fluor 594 (A-11058, ThermoFisher Scientific) was incubated at 1:200 in blocking buffer for 1-h at room temperature. Sections were washed, stained with DAPI, and coverslip mounted.

Researchers were blinded for imaging and analysis. Sections were imaged using a Multi-Laser CLSM Leica SP5 microscope (Leica Microsystems). In one retina, a minimum of three fields of view were taken per animal using the 40 × objective. To analyse extravascular albumin staining, brightfield images were used to trace around the retina and exclude choroidal and vitreous staining, using ImageJ. In the red channel, retinal area, Integrated density of the retina (IntDen_retina_), and four measurements of background mean fluorescence were taken to calculate corrected total retinal albumin fluorescence (CTF_retina_). Retinal vessel albumin staining appeared as bright circles (vessels in cross section) or long tubes (longitudinal sections of vessels). Vessels were traced around, and the area and IntDen_vascular_ were measured to calculate vascular albumin staining (CTF_vascular_). Extravascular albumin (CTF_EXV_) was defined as CTF_vascular_ subtracted from CTF_retina_. The CTF_EXV_ for each field of view was averaged for each animal.

### Statistics

Quantitative data are expressed as means ± SEM. GraphPad Prism version 5.00 used for statistical analyses (GraphPad Software, La Jolla California USA). Unpaired Student’s t-test were performed when comparing two groups. For normally distributed data, one-way ANOVA and Tukey’s multiple comparisons test was performed when comparing more than two groups and for non-normally distributed data, a Kruskal–Wallis test was performed. Statistical significance was reached when *P* < 0.05.

## Results

### HS forms part of the eGlx barrier in retinal and glomerular microvasculature

**Fig. 1:**
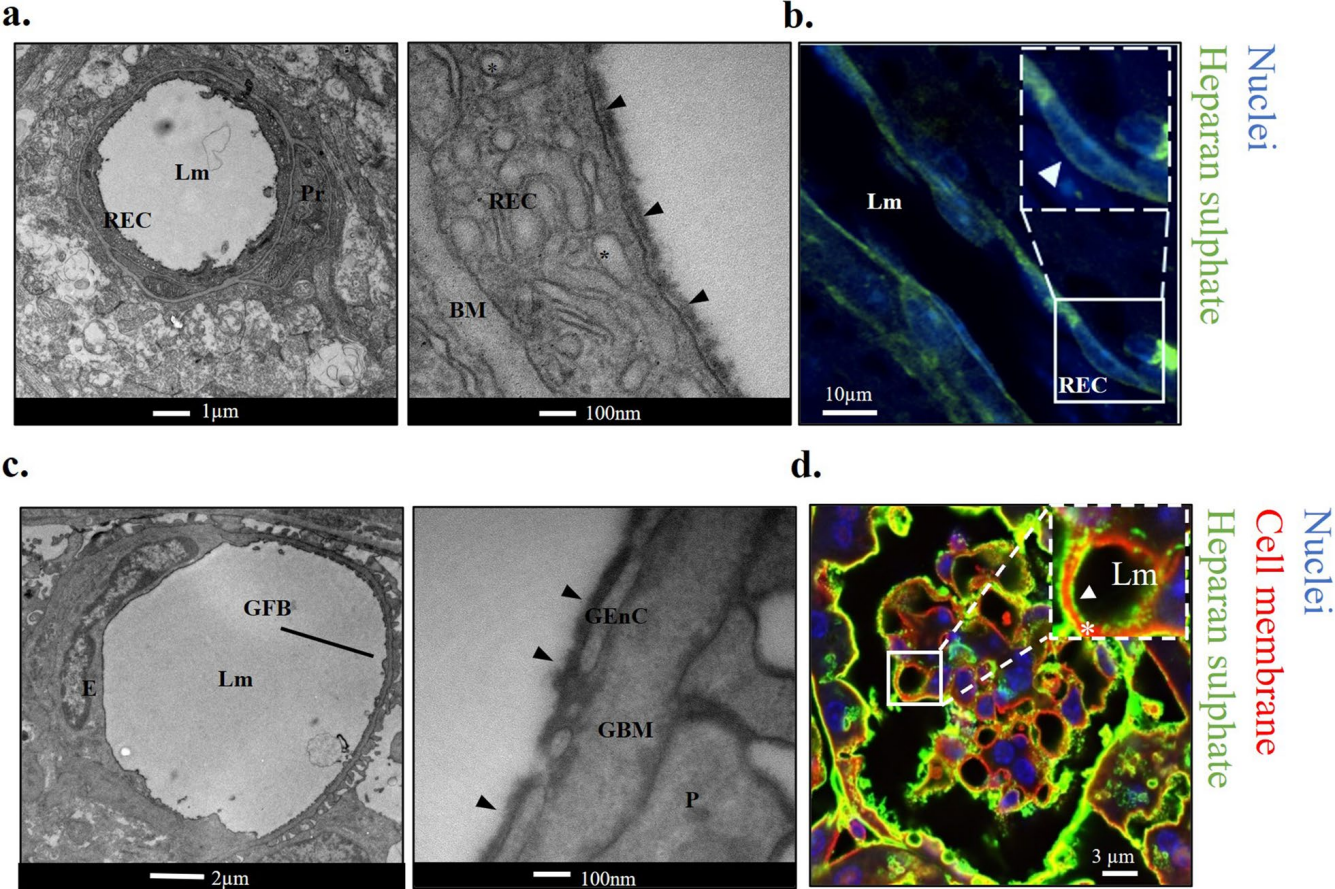
HS forms part of the eGlx barrier in retinal and glomerular microvasculature. **a** Left: low magnification representative TEM image of retinal vessel from mouse that had been cardiac perfused with Alcian blue and glutaraldehyde. Lumen (Lm), retinal endothelial cell (REC), and pericyte (Pr) indicated. Right: high magnification image showing eGlx staining on luminal side of REC (arrow heads). Basement membrane (BM) indicated. REC vesicles (*) were also visible. **b** Retinal flat mounts stained with anti-HS in green. DAPI in blue. Retinal endothelial cell (REC) and lumen (Lm) indicated. Note the green signal on luminal side of REC nucleus (arrowhead), indicating presence of HS in the eGlx. **c** Left: low magnification representative EM image of glomerular capillary from mouse that had been cardiac perfused with Alcian blue and glutaraldehyde. Lumen (Lm) and glomerular filtration barrier (GFB) indicated. Right panel: High magnification image showing eGlx staining (arrow heads) on luminal glomerular endothelial cell (GEnC). Glomerular basement membrane (GBM) and podocyte (P) indicated. **d** Mouse kidney tissue stained with anti-HS in green, DAPI in blue, membranes in red (R18). Inset shows zoomed in image of eGlx HS staining (white arrow head) on luminal (LM) side of vessel. *GBM.

We confirmed the presence of eGlx and HS on the luminal surface of both retinal and glomerular microvessels in mouse tissue using healthy C57BL/6J mice, by immunofluorescence and EM. In the retina, endothelial cells (RECs) were lined with an electron dense layer when visualised by TEM, indicating the presence of eGlx (Fig. 1a). Flat mounts revealed staining of HS in the retinal tissue, including on the luminal surface of vascular RECs, confirming the presence of HS in the eGlx (Fig. 1b). Similarly, GEnCs were lined with an electron dense layer on the luminal side of endothelial cells (Fig. 1c), consistent with eGlx staining. HS staining was present in the glomerulus as expected and was present on the luminal surface of GEnCs (Fig. 1d), indicating its presence in the eGlx.

### Enzymatic removal of HS results in reduced eGlx and loss of barrier function in the retina and glomerulus

**Fig. 2:**
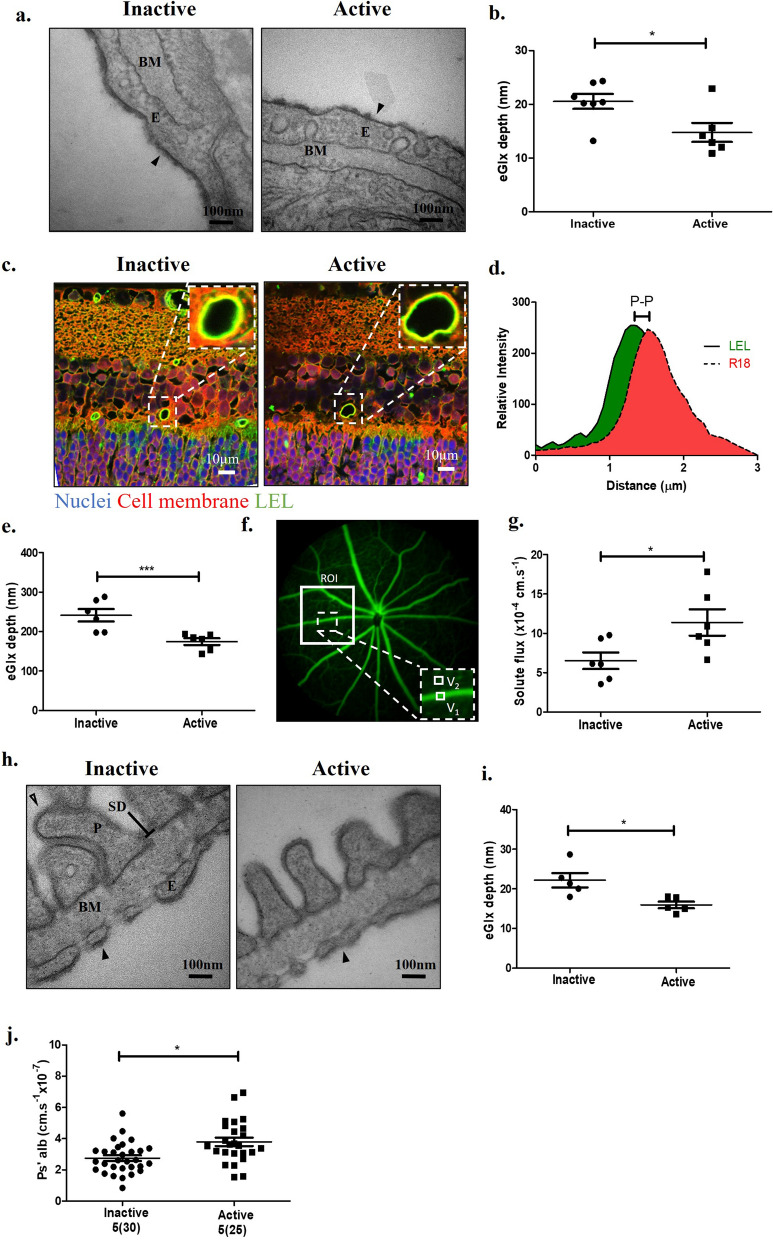
Enzymatic removal of HS results in reduced eGlx and loss of barrier function in the retina and glomerulus. **a** Representative TEM images of retinal vessels from mice perfused with inactive or active heparinase III and then Alcian Blue to visualise eGlx and glutaraldehyde for EM preparation. Basement membrane (BM) and retinal endothelial cell (E) indicated. Arrowheads point to eGlx on endothelial cell. **b** Glycocalyx depth measured. Mouse averages shown (n = 6 mice (inactive) and n = 7 mice (active), unpaired t-test indicated, *P < 0.05). **c** Representative images of lectin-stained retina sections from mice treated with inactive or active heparinase III. Lectin (FITC-LEL) staining in green, R18 cell membrane stain in red, and DAPI in blue. Inset images show green signal on the luminal side of the vessel, indicating eGlx staining. **d** Example of confocal fluorescence peak-to-peak (P-P) measurement for measurements of eGlx depth using LEL and cell membrane (R18) staining. **e** Confocal fluorescence profile peak-to-peak measurements in capillaries were taken for a minimum of three vessels per animal. Mouse averages shown (n = 6 mice/group, *P < 0.05*,* unpaired t-test indicated). **f** Image of mouse retina perfused with sodium fluorescein angiography. Within the selected ROI, the area in main vessel (V_1_) and in the tissue next to vessel (V_2_) measured over time to calculate solute flux. **g** Solute flux after treatment with inactive or active enzyme. (n = 6 mice/group, *P < 0.05*,* unpaired t-test indicated) **h** Representative glomerular filtration barrier TEM images of glomerular capillary in inactive and active heparinase III treated mice showing podocyte (P), podocyte slit diaphragm (SD), endothelial cell (E), basement membrane (BM), podocyte Glx (open arrowhead), and eGlx (solid arrowhead). **i** Quantification of EM images measuring eGlx depth (n = 5 mice/group,*P < 0.05, unpaired t-test). **j** Glomerular albumin permeability (P*s’alb*) measured for both groups. Glomeruli analysed shown on graph and in parentheses. Stats performed on mouse number (n = 5 mice/group,*P < 0.05, unpaired t-test).

To determine the systemic impact of HS removal on the eGlx barrier function in vivo, mice were given a vascular bolus (30 min) of active or inactive heparinase III, an HS cleaving enzyme. Although the presence of HS in the retinal microvasculature is well established, functional studies for HS in the retinal eGlx have not been published. We therefore examined the impact of enzymatic HS removal on retinal eGlx and barrier function. TEM analysis on Alcian blue perfused retinas showed a significant reduction in eGlx depth in active enzyme treated mice compared to inactive enzyme (Fig. 2a, b). We next stained mouse retinas with the cell membrane dye R18 and the lectin LEL, which binds to glycocalyx components in mice (Fig. 2c) with a similar staining pattern to HS (Additional file 1: Fig. S3). Using the established method of fluorescence peak-to-peak measurements (Fig. 2d), which measures the anatomic difference between peak LEL fluorescence and peak cell membrane (R18) fluorescence to measure eGlx depth [25, 36, 37], we found that mice treated with active enzyme had significantly reduced eGlx depth compared to inactive enzyme treated mice (Fig. 2e). As these results were consistent with EM analysis in the same model (current gold standard for glycocalyx visualization) we applied this staining method to all subsequent models in the retina, due to its relative simplicity. There is a notable difference in eGlx depth values between lectin peak-to-peak and TEM measurements which results from processing of tissue for TEM, involving a series of dehydration steps which cause collapse of the glycocalyx’s gel-like structure [40].

We next investigated the impact of eGlx HS shedding on retinal vascular barrier function, using a technique developed by Allen et al*.*, to measure retinal solute flux using fluorescein angiography (Fig. 2f) [38]. Active enzyme treated mice had a significantly higher retinal solute flux compared to inactive enzyme treated mice (Fig. 2g), indicating a functional role for HS in the blood-retina-barrier.

In the kidney, mice treated with active enzyme had reduced eGlx depth compared to mice treated with inactive enzyme, as determined by quantitative EM (Fig. 2h, i). In contrast, there was no measurable change in podocyte Glx (Additional file 1: Fig. S4a) or in GFB measurements (Additional file 1: Fig. S4b–e). Previous reports suggested that whole body heparanase over expression in transgenic mice does not result in proteinuria, as changes in urine albumin levels are modest, albeit significant [41]. However, albuminuria is not a sensitive measure of glomerular albumin permeability since in can be confounded by other factors including tubular reabsorption of filtered albumin [42]. To determine the direct impact of eGlx HS removal from glomerular vessels, we used an ex vivo assay previously developed by our group, which specifically measures glomerular albumin permeability in the absence of tubular reabsorption and haemodynamic confounders [39]. We found that treatment with active heparinase III significantly increased glomerular albumin permeability (P*s*_*’alb*_) compared to inactive enzyme treatment (Fig. 2j) despite a lack in GFB morphology or podocyte changes, an indication that these permeability changes are eGlx dependant. Together, these results indicate that HS is functionally important in both retinal and glomerular microvasculature.

### Conditional knockout of the HS polymerizing enzyme, Ext1, in endothelial cells leads to reduced eGlx and compromised microvascular barrier function.

**Fig. 3:**
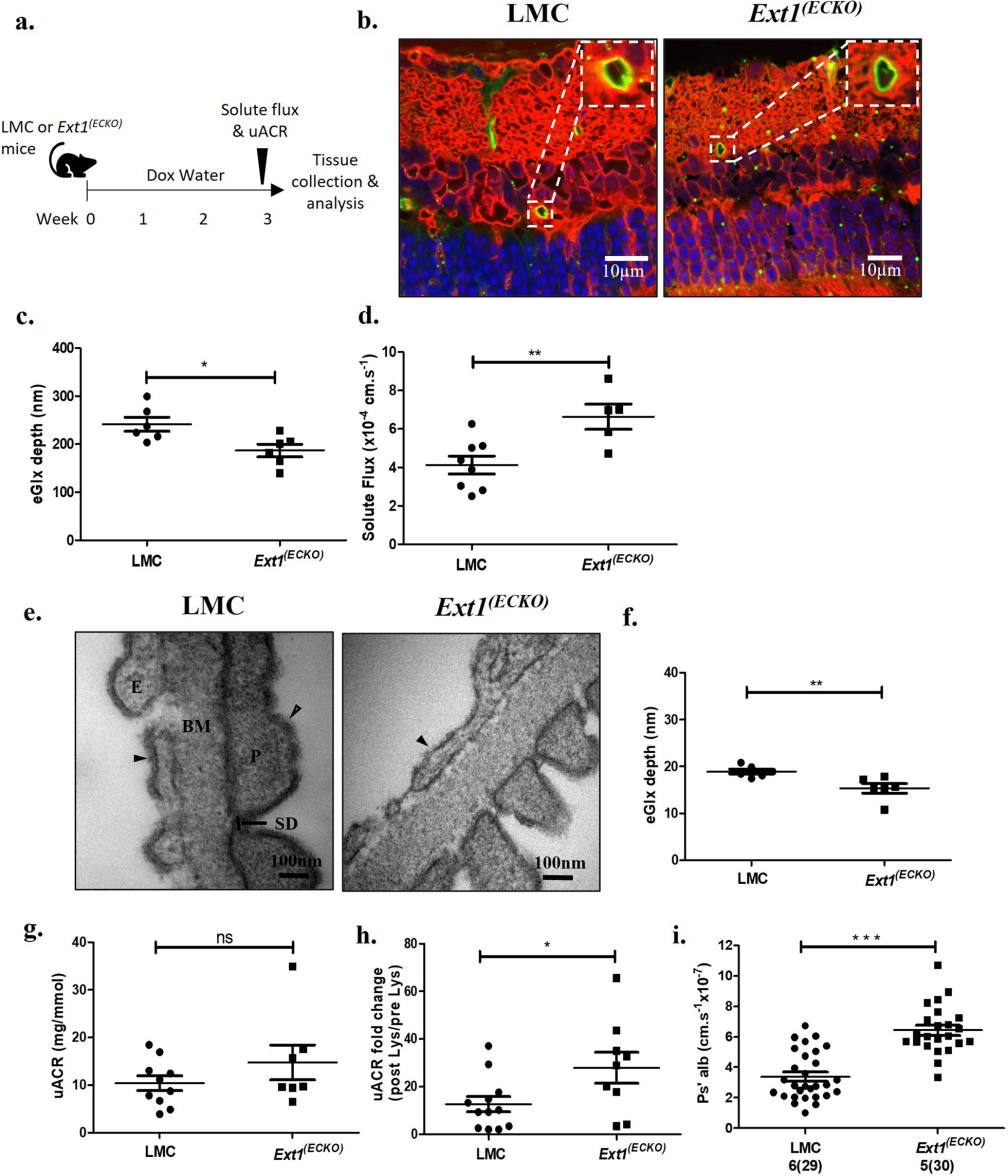
Conditional knockout of the HS polymerizing enzyme, Ext1, in endothelial cells leads to reduced eGlx and compromised microvascular barrier function. **a** Schematic of experimental timeline. Age and sex matched littermate controls (LMCs) or *Ext1* endothelial specific conditional knockout mice (*Ext1*^*(ECKO*)^) were given Doxycycline water for three weeks to induce knock-out. At three weeks, fluorescein angiographies were performed for solute flux measurements and urine analysis experiments conducted. Eye and kidney tissue was collected and analysed after three weeks of Doxycycline treatment. **b** Representative images of lectin-stained retinal tissue from LMC and *Ext1*^*(ECKO*)^ mice. Lectin staining in green, R18 cell membrane stain in red, and DAPI in blue. Inset images show green signal on luminal side of the vessel, indicating eGlx staining. **c** Confocal fluorescence profile peak-to-peak measurements in capillaries were taken for a minimum of three vessels per animal. Mouse averages shown (n = 6, *P < 0.05, unpaired t-test). **d** Solute flux measured in LMC (n = 8 mice) and *Ext1*^*(ECKO)*^ (n = 5 mice) after three weeks of doxycycline treatment (**P < 0.01, unpaired t-test). **e** Representative glomerular filtration barrier TEM images of litter mate control (LMC) and *Ext1* endothelial specific conditional knockout mice (*Ext1*^*(ECKO)*^) showing podocyte (P), podocyte slit diaphragm (SD), endothelial cell (E), basement membrane (BM), podocyte Glx (open arrow heads), and eGlx (solid arrow heads). **f** Quantification of TEM images measuring eGlx depth (n = 6 mice,**P < 0.01, unpaired t-test). **g** End point urine albumin creatinine ratios (uACR) for LMC (n = 10) and *Ext1*^*(ECKO)*^ (n = 7) mice (not significant, *P* = 0.24, unpaired t-test). **h** Fold change uACR from pre and post L-lysine (Lys) treatment in LMC (n = 12) and *Ext1*^*(ECKO)*^ (n = 9) mice (*P < 0.05, unpaired t-test). **i** Glomerular albumin permeability (P*s’alb*) measured for LMC (n = 6) and *Ext1*^*(ECKO)*^ (n = 5 mice). Number of glomeruli analysed shown on graph and in parentheses. Stats performed on mouse number (***P < 0.001, unpaired t-test).

To further confirm the role of HS in retinal and glomerular eGlx in vivo, we genetically ablated HS synthesis in an inducible endothelial specific knockout of Ext1 (*Ext1*^*(ECKO)*^ mice) mouse model (Fig. 3a). Mice had no gross retinal or glomerular changes shown by OCT and H&E staining, respectively (Additional file 1: Fig. S5a,b). We further confirmed specific reduction of eGlx HS by immunofluorescence in *Ext1*^*(ECKO)*^ mice (Additional file 1: Fig. S5c,d). We validated this model by qPCR on FACS-isolated endothelial cells and demonstrated significantly lower Ext1 mRNA in *Ext1*^*(E*CKO)^ compared to littermate controls (LMCs) (Additional file 1: Fig. S5e).

We next examined the impact of reduced endothelial HS synthesis and found a significant reduction in retinal eGlx depth in *Ext1*^*(ECKO)*^ mice compared to LMCs (Fig. 3b, c), consistent with our results when HS was removed enzymatically. Furthermore, solute flux in *Ext1*^*(ECKO)*^ mice was higher compared to LMCs (Fig. 3d), providing further confirmation of the importance of HS in the retinal eGlx.

In the kidney, quantitative analysis of TEM images (Fig. 3e, f) demonstrated reduced glomerular eGlx depth in *Ext1*^*(ECKO)*^ compared to LMCs. In contrast, podocyte Glx in the same animals was not affected (Additional file 1: Fig. S6a), nor were any other measured ultrastructural filtration barrier parameters (Additional file 1: Fig. S6b–e). No significant change in uACR was observed (Fig. 3g) in *Ext1*^*(ECKO)*^, consistent with data from multiple osteochondroma (MO) patients, an autosomal dominant disease resulting from loss of function in EXT1 or EXT2 [43]. However, smaller increases in glomerular albumin permeability can be masked by tubular reabsorption of filtered albumin, as previously described [44, 45]. To prevent this, we treated mice with the potent tubular reabsorption inhibitor l-Lysine which allowed us to unmask a significant increase in uACR fold change of 27.93 ± 6.525 in *Ext1*^*(ECKO)*^ mice compared to 12.61 ± 3.189 for LMCs (Fig. 3h) [32]. In line with these results, *Ext1*^*(ECKO)*^ mice had a significant increase in albumin permeability (P*s*_*’alb*_) measured by the more direct and sensitive ex vivo glomerular albumin permeability assay (Fig. 3i). These data are consistent with results obtained from heparinase III treated mice, further providing evidence that HS within the eGlx is an important component of the microvascular barrier, demonstrated in the eye and kidney.

### OVZ/HS-1638 treatment in db/db mice prevents systemic eGlx damage and associated microvascular permeability changes

**Fig. 4:**
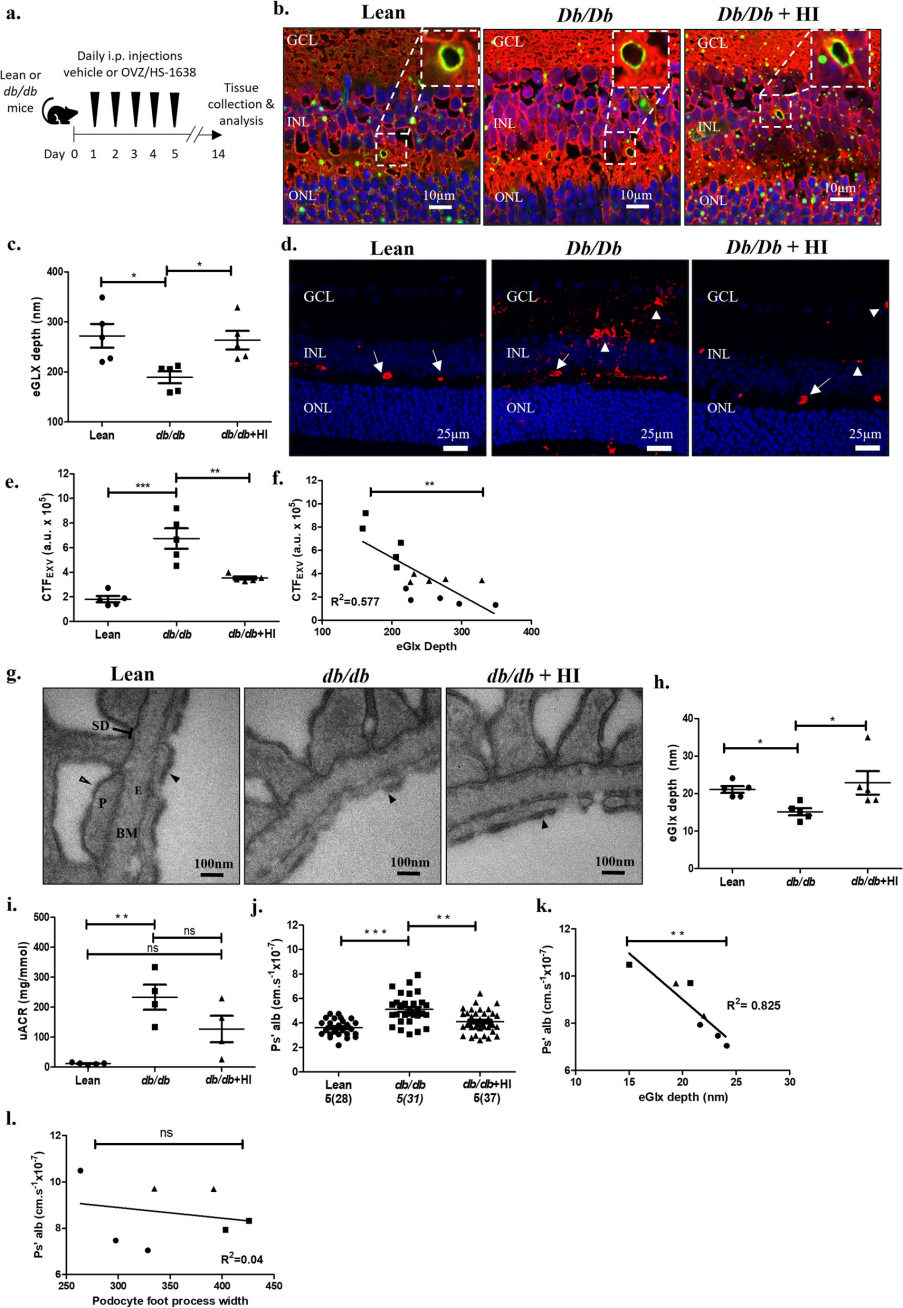
Treatment with the heparanase inhibitor OVS/HS-1638 in db/db mice prevents systemic eGlx damage and associated microvascular permeability changes. **a** Schematic of experimental timeline. Lean or db/db mice were treated with vehicle or OVZ/HS-1638 daily for 14-days by i.p. End-point urine was collected for analysis and eye and kidney tissue collected for analysis. **b** Retinas from lean and *db/db* mice treated with vehicle (*db/db)* or with OVZ/HS-1638 (*db/db* + HI) were stained with FITC-LEL (green), R18 cell membrane stain (red) and DAPI (blue). Inset images show green staining on luminal side of vessel, indicating eGlx staining. **c** Glycocalyx depth in lean and diabetic animals was measured using confocal fluorescence profile peak-to-peak (n = 5, mice per group, *P ≤ 0.05*,* One-way ANOVA with Tukey’s multiple comparison test). **d** Retinas from lean and *db/db* mice treated with vehicle (*db/db*) or with OVZ/HS-1638 (*db/db* + HI) were stained for albumin (red) and DAPI (blue). Arrows point to vessels filled with albumin and arrowheads point to extravascular albumin. Extravascular albumin staining found near Ganglion cell layer (GCL), inner nuclear layer (INL), and outer nuclear layer (ONL) in *db/db* retina. **e** Extravascular corrected total cell fluorescence (CTF_EXV_) in arbitrary units (a.u.) (n = 5 mice per group, **P ≤ 0.01, ***P ≤ 0.001*,* One-way ANOVA with Tukey’s multiple comparison test). **f** Average CTF_EXV_ value for each mouse was plotted against corresponding eGlx depth for same animal (n = 15 total mice, **P ≤ 0.01*,* Pearson correlation analysis). **g** Representative TEM images of lean, diabetic (*db/db*) and diabetic mice treated with OVZ/HS-1638 (*db/db* + HI) mouse glomerular filtration barrier showing podocyte (P), podocyte slit diaphragm (SD), endothelial cell (E), basement membrane (BM), podocyte Glx (open arrow heads), and eGlx (solid arrow heads). **h** Quantification of TEM images measuring eGlx depth (n = 5 mice,*P < 0.05, Kruskal–Wallis test). **i** End point urine albumin creatinine ratio for lean (n = 5), *db/db* (n = 4), and *db/db* + HI (n = 4) (**P < 0.01, Kruskal–Wallis test). **j** Glomerular albumin permeability (P*s’alb*) measured. Glomeruli analysed shown on graph and in parentheses. Stats performed on mouse number (n = 5, **P < 0.01, ***P < 0.001, One way ANOVA, Tukey’s multiple comparison test). (k,l) Correlation analysis. (k) P*s’alb* vs eGlx depth (n = 7,**P < 0.01), (l) Ps’*alb* vs podocyte foot process width (n = 7, *P* > 0.05, not significant (ns)).

The role of HS in the retinal eGlx has never been demonstrated, and our HS depletion models demonstrate a common functional role for HS in the eGlx in both the retinal and glomerular microvasculature. We next investigated the impact of eGlx damage in diabetes and determined whether treatment with the heparanase inhibitor OVZ/HS-1638 could prevent systemic eGlx damage and the associated permeability increases in a T2D mouse model using *db/db* mice (Fig. 4a). *Db/db* mice have previously been shown to express elevated levels of heparanase [46]. Although OVZ/HS-1638 is already known to have heparanase inhibitor activity [29], we confirmed OVZ/HS-1638 could effectively inhibit HS removal from the eGlx by heparanase in vitro (Additional file 1: Fig. S7a, b). Nine-week-old *db/db* mice were hyperglycemic and obese at the start of the study compared to control *db/* + (lean) mice and remained so for the duration of the study (Table 1).

**Table 1:** Blood glucose and body weights for lean (*db*/ +), diabetic (*db/db*) and *db/db* plus OVZ/HS-1638 (HI) mice from 9–11week age

	Age (week)	Db/ + (n = 5)	Db/db (n = 5)	Db/db + HI (n = 5)
Blood glucose (mmol/L) ± SEM	9	8.0 ± 0.5	23.4 ± 1.6***	20.6 ± 0.8***
	10	7.5 ± 0.4	25.9 ± 1.7***	25.2 ± 1.0***
	11	7.9 ± 0.3	30.3 ± 0.9***	30.3 ± 0.8***
Body weight (g) ± SEM	9	27.7 ± 0.3	42.0 ± 1.0***	42.8 ± 0.03***
	10	28.2 ± 0.5	44.1 ± 0.4***	45.1 ± 1.1***
	11	29.0 ± 0.5	46.3 ± 0.3***	47.0 ± 1.4***

*SEM*  standard error of the mean

Two-way ANOVA with Bonferroni post-hoc tests indicated

***p < 0.001 compared to lean mice condition

No significant differences were seen between db/db and db/db + HI

In the retina, eGlx depth was reduced in *db/db* mice compared to lean, however, this difference was not present in db/db mice treated with OVZ/HS-1638 (Fig. 4b, c). These results demonstrate that retinal eGlx structure is affected by diabetes and can be protected by prevention of HS degradation. To determine whether preservation of the eGlx protected against retinal vascular leakage, we analysed levels of extravascular albumin in the retina. As albumin does not cross the blood-retina-barrier under physiological conditions, the quantification of extravascular albumin reflects cumulative retinal vascular leakage over time, and has been previously used to determine localization of vascular leak in human post mortem tissue [47–49]. We used immunolabelling of extravascular albumin as an indicator of vascular permeability rather than solute flux due to substantial differences in the rate of fluorescein uptake between lean and *db/db* mice, which makes measurements of solute flux by fluorescein angiography unreliable in obese mouse models of diabetes. We found that extravascular albumin had accumulated in the retinal tissue of *db/db* mice, whereas albumin remained within the retinal blood vessels of lean mice (Fig. 4d, e). This difference in albumin staining was not due to any differences in retinal blood vessel density between groups (Additional file 1: Fig. S8). Furthermore, the amount of extravascular albumin in the retinal tissue was significantly lower in OVZ/HS-1638 treated mice (Fig. 4d, e). Moreover, increased levels of extravascular albumin in retinal tissue were linearly correlated with reduced eGlx depth (Fig. 4f).

We next investigated if protection of eGlx structure and barrier function with OVZ/HS-1638 treatment extended to the glomerulus, in the same animals. As in the retina, glomerular eGlx depth was significantly reduced in *db/db* mice compared to lean mice but this difference was not present in *db/db* mice treated with OVZ/HS-1638 (*db/db* + HI) (Fig. 4g, h). Podocyte Glx measurements were unchanged across the three groups (Additional file 1: Fig. S9a) and no significant changes were observed in basement membrane thickness or endothelial fenestration density (Additional file 1: Fig. S9b, c). A reduction in podocyte slit diaphragm width and an increase in podocyte foot process width was observed in *db/db* mice (Additional file 1: Fig. S9d, e), suggesting podocyte foot process effacement as anticipated with a DKD phenotype. Importantly, these changes in podocyte morphology were not affected by OVZ/HS-1638 treatment. Glomerular PAS staining, a measure of mesangial expansion [50] was significantly lower in inhibitor treated *db/db* mice, suggesting OVZ/HS-1638 also protects against this feature of DKD (Additional file 1: Fig. S10).

We next measured uACR to assess the effect of OVZ/HS-1638 treatment on GFB function. We found that *db/db* mice had significantly higher uACR compared to lean mice, while *db/db* + HI mice did not (Fig. 4i). Similarly, *db/db* mice had an increase in P*s*_*’alb*_ compared to lean mice, whilst permeability in *db/db* + HI was significantly reduced compared to *db/db* mice, and restored to similar levels as lean mice (Fig. 4j). Furthermore, mice in which both eGlx measurements and permeability were measured, revealed a significant correlation between eGlx depth versus P*s*_*’alb*_ (Fig. 4k). In contrast, no significant correlation between podocyte foot process width and P*s*_*’alb*_ in the same mice was observed (Fig. 4l), indicating that the changes we observed in glomerular permeability are not dependant on podocyte morphology. Together, these data demonstrate that systemic treatment with OVZ/HS-1638 can simultaneously protect the eGlx of both the retina and glomerulus in *db/db* mice and prevent the pathological microvascular permeability changes typically associated with diabetes.

## Discussion

Our results demonstrate that microvascular complications in diabetes can be treated systemically, by targeting HS in the eGlx to restore vascular barrier properties. Microvascular permeability changes occur early in DR and DKD, and are predictors of disease progression [4, 51]. We show that HS is a key component of the microvascular barrier, using the retina and glomerulus as examples. In this study, we used two independent methods to deplete HS from the eGlx in vivo: in the first, mice were treated with heparinase III, and in the second, the HS polymerase component Exotosin-1 was conditionally knocked out from endothelial cells in *Ext1*^*(ECKO)*^ mice. Our results showed that HS depletion by either method resulted in reduced retinal and glomerular eGlx depth. Furthermore, both methods resulted in greater retinal solute flux and glomerular permeability, indicating the importance of HS to eGlx barrier function in two different vascular beds. Importantly, our data provided further evidence that protection of the eGlx using a novel class of heparanase inhibitors OVZ/HS-1638, is an effective systemic approach to protect against the microvascular complications in diabetes. OVZ/HS-1638 holds competitive advantages over commercially available inhibitors, including lack of anticoagulant activity, increased stability, and cheaper/less complex synthesis [29], making this class of heparanase inhibitor an excellent candidate for clinical use.

While HS is abundant throughout the retina [52], to date, there have been no studies published demonstrating the role of eGlx HS in the retina. Previously, Witmer et al*.* investigated HS in the vascular basement membrane of postmortem retina tissue from diabetic patients. The authors suggested that increased microvascular permeability, measured by PAL-E staining [53], was not associated with changes in retinal basement membrane HS or its basement membrane proteoglycans agrin and perlecan [54]. This is in line with studies on mice lacking perlecan and agrin in the glomerular basement membrane, which have no changes in GFB function or structure [55]. These studies suggest that basement membrane HS does not contribute to microvascular barrier function. In contrast, our data shows clear changes in barrier function when HS within the eGlx is depleted in vivo, demonstrating an important role of HS within the eGlx in the retinal microvasculature, not previously shown.

The systemic role of HS within the eGlx was supported by our heparinase III model and *Ext1*^*(ECKO)*^ mouse models, in which depletion of HS resulted in higher glomerular albumin permeability [43]. In health, small amounts of albumin which are filtered through the GFB are almost entirely reabsorbed by the tubules [56]. It is only when the system is overwhelmed by increasing glomerular leakage of albumin in disease, that changes in albuminuria are observed. We did not observe significant changes in uACR in *Ext1*^*(ECKO)*^ mice. Yet, we found that when we inhibited tubular reabsorption with _L_-Lysine, there was a large fold increase in the uACRs in *Ext1*^*(ECKO)*^ mice compared to LMCs, in agreement with our ex vivo glomerular albumin permeability results which measures permeability in the absence of tubular reabsorption. Contrary to previous literature which have used albumin clearance *i.e.* uACR, and not glomerular albumin permeability, our data provides clear evidence that depleting HS from the glomerular eGlx is sufficient to negatively impact glomerular barrier function. Microalbuminuria is a predictor of progressive renal disease [57], so factors that causes moderate increases in microalbuminuria are important to consider in the context of diabetes and the early management of DKD. We also showed that both enzymatic and genetic depletion of HS did not alter GFB structures other than the eGlx, including glomerular basement membrane thickness, podocyte foot process and slit diaphragm width, and endothelial cell fenestrations, indicating that the functional changes we observed were specifically caused by structural changes to the glycocalyx.

Current treatments for diabetic microvascular complications are designed to treat the specific local complication (i.e., DR or DKD and not both), they are not preventative, and they each have possible risks. For example, retinal laser photocoagulation therapy for DME can result in scotomas, reduced visual acuity and subretinal fibrosis [14], and anti-VEGFA therapies may lead to proteinuria, compounding kidney injury in those already at risk for DKD [58, 59]. Furthermore, DKD is still progressive, despite the potential renoprotective effects of treatments such as SGLT2i and RAS inhibitors. Therefore, there is a clinical need for treatments that can prevent the development and progression of DR and DKD.

Heparanase has gained interest as a druggable target in some diseases that are associated with chronic inflammation, such as cancer and diabetes. In line with our results presented here in diabetes, Gil. et al. demonstrated that the heparanase inhibitor SST0001 reduced albuminuria in T2D mice and further determined that heparanase upregulation in DKD results from activation of the heparanase promoter by the transcription factor early growth response-1 (Egr1) [60]. As indicated by the authors, although protective effects of heparanase inhibition were shown in diabetic mice, a limitation of the study was that the findings were not sufficient to determine the mode of heparanase action in DKD pathogenesis [60]. Using our novel heparanase inhibitor OVZ/HS-1638, we have now demonstrated that heparanase acts on the eGlx, as inhibiting heparanase protects the eGlx and prevents microvascular permeability changes. We showed that OVZ/HS-1638 treatment of *db/db* mice protected the retinal eGlx and reduced albumin leak in the retina. In the same mice, we also showed OVZ/HS-1638 treatment prevented glomerular eGlx damage and reduced glomerular permeability. The clinical relevance to patients is that HS in the eGlx of different vascular beds can be therapeutically targeted with a systemic medication, thereby preventing the microvascular permeability changes associated with DR and DKD.

In health, heparanase expression is tightly regulated; it is expressed only in a subset of tissues, mainly placenta and skin [61, 62]. Heparanase expression and activity is largely modulated by pathophysiological conditions, such as pH, as heparanase is essentially inactive in neutral conditions [63]. This increases the attractiveness of heparanase as a drug target. Heparanase inhibitors have been shown to slow disease progression in cancer studies; the most promising and potent inhibitor in clinical trials is PG545, a polysulfated oligosaccharide-cholestanyl aglycone [60, 64, 65]. Despite this, no heparanase inhibitors have been approved for clinical use as they are limited by cost, production complexity, and off target effects such as anticoagulant activity and thrombocytopenia resulting from their structural similarity to heparin [29, 66, 67]. Unlike other heparanase inhibitors currently in clinical trials, such as SST0001 and PG545 which are all oligo-/polysaccharide derivatives, OVZ/HS-1638 is a polyvalent dendrimer, allowing for multiple heparanase interactions at multiple points [29, 66]. The heterogenous nature of oligo-/polysaccharide derivatives such as PG545 also results in batch variations, further complicating FDA approval. In contrast, dendrimer synthesis yields single entity compounds, which means OVS/HS-1638 preparations are homogenous. Previous in vitro experiments show that OVZ/HS-1638 inhibits heparanase at nanomolar concentrations (IC_50_ 11 nM) and displays no anticoagulant activity toward Factor Xa, even at concentrations > 100-fold than the IC_50_ for heparanase inhibition [29], indicating the highly specific nature of this inhibitor. Computer modelling of these polyvalent dendrimers suggest they bind to heparanase in the same region as HS with a greater affinity, indicating OVZ/HS-1638 may act as a competitive inhibitor [68]. In addition to the forementioned competitive advantages, OVZ/HS-1638 inhibits angiogenesis [29], and was recently shown to rescue blood–brain-integrity in a model of neuroinflammation [69]. These results indicate OVZ/HS-1638 holds significant clinical promise. Further studies on pharmacokinetics, bioavailability, and safety of OVZ/HS-1638 are in progress, to support its potential for clinical evaluation [29].

## Limitations

To our knowledge, this study is the first to demonstrate systemic prevention of microvascular complications associated with diabetes, demonstrated in the eye and kidney. However, there are still limitations which remain. Firstly, our diabetic study lacked a group in which lean non-diabetic mice were treated with OVZ/HS-1638. At the time this study was designed, quantities of this inhibitor were limited due to small scale synthesis. However, this inhibitor has been used in other in vivo disease models such as myeloma, colorectal cancer, osteogenesis, as well as multiple sclerosis [29, 68–70]. As in our study, treatment with OVZ/HS-1638 was well tolerated, with no apparent adverse effects observed. Additionally, we did not observe any increased negative effects in our treated *db/db* mice in the parameters measured, but we cannot be certain that OVZ/HS-1638 does not have any effect in healthy mice. The forementioned pharmacokinetic, bioavailability, and safety studies using this inhibitor will be conducted in non-diseased models, and therefore any effect of OVZ/HS-1638 in healthy mice will be determined. Along with these studies, it will be informative to measure heparanase activity in *db/db* mice treated with OVZ/HS-1638. In addition to the previously discussed heparanase activity studies by Zubkova et al*.* 2018, our in vitro work in endothelial cells which confirmed inhibition of HS shedding by heparanase in OVZ/HS-1538 treated cells, gives us full confidence in OVZ/HS-1638’s ability to inhibit heparanase activity. Nevertheless, studies which confirm reduced heparanase activity in vivo will add value to our ongoing work as we progress this inhibitor toward clinical use. Another limitation of this study is the time of treatment in *db/db* mice. Diabetic mice began treatment at 9-weeks-of-age, a relatively early stage of disease progression for DR and DKD in this model. It is likely that in a clinical setting, such treatments would be given at a wide range of disease stages (early and late). Although *db/db* mice were significantly hyperglycemic and obese at the time of the treatment, we cannot conclude how this inhibitor would perform when given at a more advanced stage of disease. Ongoing research in our group will address this question and will be of great clinical relevance.

## Conclusion

In conclusion, our work demonstrates that HS is an important systemic contributor to microvascular permeability function, demonstrated in two structurally unique microvascular beds, the retina and glomerulus. Furthermore, we have shown that protecting the eGlx with a novel polyvalent dendrimer which acts as a heparanase inhibitor, can prevent systemic microvascular permeability changes typically caused by diabetes. Our focus, in future work, is to achieve a pre-clinical experimental and safety package for OVZ/HS-1638 for treatment of DKD and DR in clinics.

### Supplementary Information

**Additional file 1: Figure S1.** Measurements of glomerular filtration parameters and glycocalyx depth. **a** Area of measurements (dotted lines) for podocyte foot process (PFP) width, slit diagraph (SD) width, basement membrane (BM) thickness, and length of BM. Number of fenestrations were counted as indicated, and divided by the length of BM to obtain fenestrations/µm. **b** For eGlx measurements, a grid was overlayed over the image. Red arrows point to examples of eGlx staining. Measurements were taken where the intersecting grid lines intersected the lipid bilayer, examples indicated by (x) and measurement example indicated by dotted lines on inset image. **Figure S2.** Structure of heparanase inhibitor OVZ/HS-1638. OVZ/HS-1638 is a single entity chemical polyvalent dendrimer which allows for multiple target interactions at multiple points on the inhibitor [29]. **Figure S3**. LEL stains eGlx components in the retina. Mouse retinal vessel on retinal flat mount stained with FITC-LEL (green) and DAPI (blue). Lumen (Lm) of vessel indicated. Arrow points to eGlx staining on surface of endothelial cell. **Figure S4.** A vascular bolus of heparinase III has no impact on podocyte glycocalyx or other ultrafiltration parameters. Glomerular filtration barrier measurements in mice treated with inactive or active heparinase III. **a** Podocyte glycocalyx depth. **b** Basement membrane (BM) thickness. **c** Fenestration density. **d** Slit diaphragm width. **e** Podocyte foot process width. No statistically significant differences found (n = 5 mice, unpaired t- test, not significant). **Figure S5.** *Ext1*^*(ECKO)*^ mice have no gross changes in retinal or glomerular morphology and have reduced eGlx HS. **a** Representative optical coherence tomography (OCT) images of retinas from LMC and *Ext1*^*(ECKO)*^ mice, showing no changes in morphology. Scale bar 50 µm. **b** Representative images of cortex Haematoxylin and Eosin staining at × 10 and × 40 magnification are shown for LMC and *Ext1*^*(ECKO)*^ mice, showing no change in gross morphology. **c** Representative images of heparan sulphate staining on kidney from LMC and *Ext1*^*(ECKO)*^ mice. Inset shows eGlx heparan sulphate staining (green) on luminal side of capillary (arrowhead). Membrane stain R18 (red) used to stain cell membranes and DAPI for nuclear staining (blue). Red blood cells (*) and basement membrane (BM) indicated. Scale bar = 20 µm **d** Analysis of glomerular capillary eGlx heparan sulphate staining. (n = 6 mice, **p < 0.01, unpaired t-test). **e** Quantitative PCR performed on FAC sorted glomerular endothelial cells from littermate control (LMC) and *Ext1*^*(ECKO)*^ mice. (n = 3 mice, **p < 0.01, unpaired t-test). **Figure S6.** *Ext1*^*(ECKO)*^ mice have no change in podocyte glycocalyx or other ultrafiltration parameters. Glomerular filtration barrier measurements in littermate control (LMC) and *Ext1*^*(ECKO)*^ mice. **a** Podocyte glycocalyx depth. **b** Basement membrane (BM) thickness. **c** Fenestration density. **d** Slit diaphragm width. **e** Podocyte foot process width (n = 6 mice/group, unpaired t- test, not significant). **Figure S7.** Treatment with the heparanase inhibitor OVZ/HS-1638 in vitro prevents heparan sulphate shedding by heparanase. **a** Representative confocal images of control, heparanase (HPSE), or HPSE + OVZ/HS-1638 inhibitor (HPSE + HI) treated GEnCs stained with anti-heparan sulphate in green. **b** Quantification of total heparan sulphate staining in Control, HPSE, and HPSE + HI treated GEnCs, normalized to cell number (n = 6 technical repeats, P* < 0.05,**P < 0.01, One way ANOVA for normally distributed data, Tukey’s multiple comparison test). **Figure S8.** Vessel number is not significantly impacted in *db/db* mice at 11-weeks-of-age Average number of vessels counted per animal in albumin-stained retina per field of view (FOV), minimum of three FOV counted per animal (n = 6 mice/group, One-way ANOVA with Tukey’s multiple comparison test. Not significant). **Figure S9.** Db/db mice develop early signs of diabetic nephropathy. Glomerular filtration barrier measurements for lean, diabetic (*db/db*) and diabetic mice treated with OVZ/HS-1638 (*db/db* + HI). **a** Podocyte glycocalyx depth. **b** Basement membrane (BM) thickness. **c** Fenestration density. **d** Slit diaphragm width. **e** Podocyte foot process width (n = 5 mice/group. One way ANOVA for normally distributed data, Tukey’s multiple comparison test. No statistically significant differences found for (a–b). For (d, e), *P < 0.05, **P < 0.01). **Figure S10.** Treatment with OVZ/HS-1638 prevents glycogen deposition in diabetic nephropathy in *db/db* mice. **a** Representative Periodic acid-Schiff (PAS) staining images for lean, diabetic (*db/db*) and diabetic treated with OVZ/HS-1638 (*db/db* + HI) **b** PAS staining analysed to measure fibrosis within glomerulus in lean, diabetic (*db/db*) and diabetic treated with OVZ/HS-1638 (*db/db* + HI) mice. (n = 5 mice/group, *P < 0.05 **P < 0.01, One way ANOVA for normally distributed data, Tukey’s multiple comparison test).

## Data Availability

All data and materials in this study are presented in the published article and Additional material. Data, materials, and additional information is available from the corresponding author on request.

## Abbreviations

Db/Db: BKS.Cg-+*Lepr*^*db*^*/*+*Lepr*^*db*^/OlaHsd diabetic mice
DR: Diabetic retinopathy
DKD: Diabetic kidney disease
DME: Diabetic macular oedema
eGlx: Endothelial glycocalyx
GAG: Glycosaminoglycan
GCL: Ganglion cell layer
GEnC: Glomerular endothelial cell
GFB: Glomerular filtration barrier
HI: Heparanase inhibitor
HS: Heparan sulphate
INL: Inner nuclear layer
LEL: Lycopersicon esculentum lectin
LMC: Litter mate control
ONL: Outer nuclear layer
PAS: Periodic acid-Schiff
P*s*_*’alb*_: Glomerular albumin permeability
TEM: Transmission electron microscopy
R18: Rhodamine B Chloride
uACR: Urine albumin creatinine ratio
T2D: Type 2 diabetes

## References

[1] Gheith O, Farouk N, Nampoory N, Halim MA, Al-Otaibi T (2015). Diabetic kidney disease: world wide difference of prevalence and risk factors. J Nephropharmacol.

[2] Teo ZL, Tham YC, Yu M, Chee ML, Rim TH, Cheung N (2021). Global prevalence of diabetic retinopathy and projection of burden through 2045: systematic review and meta-analysis. Ophthalmology.

[3] Verma S, Hussain ME (2017). Obesity and diabetes: an update. Diabetes Metab Syndr.

[4] Duh EJ, Sun JK, Stitt AW (2017). Diabetic retinopathy: current understanding, mechanisms, and treatment strategies. JCI Insight..

[5] Xue R, Gui D, Zheng L, Zhai R, Wang F, Wang N (2017). Mechanistic insight and management of diabetic nephropathy: recent progress and future perspective. J Diabetes Res.

[6] Dinneen SF, Gerstein HC (1997). The association of microalbuminuria and mortality in non-insulin-dependent diabetes mellitus. A systematic overview of the literature. Arch Intern Med.

[7] Allen KV, Walker JD (2003). Microalbuminuria and mortality in long-duration type 1 diabetes. Diabetes Care.

[8] Klein R, Zinman B, Gardiner R, Suissa S, Donnelly SM, Sinaiko AR (2005). The relationship of diabetic retinopathy to preclinical diabetic glomerulopathy lesions in type 1 diabetic patients: the renin-angiotensin system study. Diabetes.

[9] Kotlarsky P, Bolotin A, Dorfman K, Knyazer B, Lifshitz T, Levy J (2015). Link between retinopathy and nephropathy caused by complications of diabetes mellitus type 2. Int Ophthalmol.

[10] Lee WJ, Sobrin L, Lee MJ, Kang MH, Seong M, Cho H (2014). The relationship between diabetic retinopathy and diabetic nephropathy in a population-based study in Korea (KNHANES V-2, 3). Invest Ophthalmol Vis Sci.

[11] Yamanouchi M, Mori M, Hoshino J, Kinowaki K, Fujii T, Ohashi K (2019). Retinopathy progression and the risk of end-stage kidney disease: results from a longitudinal Japanese cohort of 232 patients with type 2 diabetes and biopsy-proven diabetic kidney disease. BMJ Open Diabetes Res Care.

[12] Group UPDS (UKPDS) (1998). Intensive blood-glucose control with sulphonylureas or insulin compared with conventional treatment and risk of complications in patients with type 2 diabetes (UKPDS 33). Lancet.

[13] Stitt AW, Curtis TM, Chen M, Medina RJ, McKay GJ, Jenkins A (2016). The progress in understanding and treatment of diabetic retinopathy. Prog Retin Eye Res.

[14] Everett LA, Paulus YM (2021). Laser therapy in the treatment of diabetic retinopathy and diabetic macular edema. Curr Diab Rep.

[15] Fong DS, Aiello L, Gardner TW, King GL, Blankenship G, Cavallerano JD (2004). Retinopathy in diabetes. Diabetes Care.

[16] Selby NM, Taal MW (2020). An updated overview of diabetic nephropathy: diagnosis, prognosis, treatment goals and latest guidelines. Diabetes Obes Metab.

[17] Salmon AH, Satchell SC (2012). Endothelial glycocalyx dysfunction in disease: albuminuria and increased microvascular permeability. J Pathol.

[18] Reitsma S, Slaaf DW, Vink H, Van Zandvoort MA, oude Egbrink MG (2007). The endothelial glycocalyx: composition, functions, and visualization. Pflugers Arch.

[19] Zeng Y, Waters M, Andrews A, Honarmandi P, Ebong EE, Rizzo V (2013). Fluid shear stress induces the clustering of heparan sulfate via mobility of glypican-1 in lipid rafts. Am J Physiol Heart Circ Physiol.

[20] Constantinescu AA, Vink H, Spaan JAE (2003). Endothelial cell glycocalyx modulates immobilization of leukocytes at the endothelial surface. Arterioscler Thromb Vasc Biol.

[21] Nieuwdorp M, van Haeften TW, Gouverneur MCLG, Mooij HL, van Lieshout MHP, Levi M (2006). Loss of endothelial glycocalyx during acute hyperglycemia coincides with endothelial dysfunction and coagulation activation in vivo. Diabetes.

[22] Qu J, Cheng Y, Wu W, Yuan L, Liu X (2021). Glycocalyx impairment in vascular disease: focus on inflammation. Front Cell Dev Biol.

[23] Duncan G, McCormick C, Tufaro F (2001). The link between heparan sulfate and hereditary bone disease: finding a function for the EXT family of putative tumor suppressor proteins. J Clin Invest.

[24] Manon-Jensen T, Itoh Y, Couchman JR (2010). Proteoglycans in health and disease: the multiple roles of syndecan shedding. FEBS J.

[25] Ramnath RD, Butler MJ, Newman G, Desideri S, Russell A, Lay AC (2020). Blocking matrix metalloproteinase-mediated syndecan-4 shedding restores the endothelial glycocalyx and glomerular filtration barrier function in early diabetic kidney disease. Kidney Int.

[26] van den Hoven MJ, Rops AL, Bakker MA, Aten J, Rutjes N, Roestenberg P (2006). Increased expression of heparanase in overt diabetic nephropathy. Kidney Int.

[27] Zhao Y, Liu J, Ten S, Zhang J, Yuan Y, Yu J (2017). Plasma heparanase is associated with blood glucose levels but not urinary microalbumin excretion in type 2 diabetic nephropathy at the early stage. Ren Fail.

[28] El-Asrar AMA, Alam K, Nawaz MI, Mohammad G, den Eynde KV, Siddiquei MM (2015). Upregulated expression of heparanase in the vitreous of patients with proliferative diabetic retinopathy originates from activated endothelial cells and leukocytes. Invest Ophthalmol Vis Sci.

[29] Zubkova OV, Ahmed YA, Guimond SE, Noble SL, Miller JH, Alfred Smith RA (2018). Dendrimer heparan sulfate glycomimetics: potent heparanase inhibitors for anticancer therapy. ACS Chem Biol.

[30] Inatani M, Irie F, Plump AS, Tessier-Lavigne M, Yamaguchi Y (2003). Mammalian brain morphogenesis and midline axon guidance require heparan sulfate. Science.

[31] Oltean S, Qiu Y, Ferguson JK, Stevens M, Neal C, Russell A (2015). Vascular endothelial growth factor-A165b is protective and restores endothelial glycocalyx in diabetic nephropathy. J Am Soc Nephrol.

[32] Kobayashi H, Yoo TM, Kim IS, Kim MK, Le N, Webber KO (1996). L-lysine effectively blocks renal uptake of 125I- or 99mTc-labeled anti-Tac disulfide-stabilized Fv fragment. Cancer Res.

[33] Jenniskens GJ, Oosterhof A, Brandwijk R, Veerkamp JH, van Kuppevelt TH (2000). Heparan sulfate heterogeneity in skeletal muscle basal lamina: demonstration by phage display-derived antibodies. J Neurosci.

[34] Dennissen MABA, Jenniskens GJ, Pieffers M, Versteeg EMM, Petitou M, Veerkamp JH (2002). Large, tissue-regulated domain diversity of heparan sulfates demonstrated by phage display antibodies*. J Biol Chem.

[35] Thompson SM, Fernig DG, Jesudason EC, Losty PD, van de Westerlo EMA, van Kuppevelt TH (2009). Heparan sulfate phage display antibodies identify distinct epitopes with complex binding characteristics. J Biol Chem.

[36] Crompton M, Ferguson JK, Ramnath RD, Onions KL, Ogier AS, Gamez M (2023). Mineralocorticoid receptor antagonism in diabetes reduces albuminuria by preserving the glomerular endothelial glycocalyx. JCI Insight.

[37] Butler MJ, Ramnath R, Kadoya H, Desposito D, Riquier-Brison A, Ferguson JK (2019). Aldosterone induces albuminuria via matrix metalloproteinase–dependent damage of the endothelial glycocalyx. Kidney Int.

[38] Allen CL, Malhi NK, Whatmore JL, Bates DO, Arkill KP (2020). Non-invasive measurement of retinal permeability in a diabetic rat model. Microcirculation.

[39] Desideri S, Onions KL, Qiu Y, Ramnath RD, Butler MJ, Neal CR (2018). A novel assay provides sensitive measurement of physiologically relevant changes in albumin permeability in isolated human and rodent glomeruli. Kidney Int.

[40] Ebong EE, Macaluso FP, Spray DC, Tarbell JM (2011). Imaging the endothelial glycocalyx in vitro by rapid freezing/freeze substitution transmission electron microscopy. Arterioscler Thromb Vasc Biol.

[41] van den Hoven MJ, Wijnhoven TJ, Li JP, Zcharia E, Dijkman HB, Wismans RG (2008). Reduction of anionic sites in the glomerular basement membrane by heparanase does not lead to proteinuria. Kidney Int.

[42] Lazzara MJ, Deen WM (2007). Model of albumin reabsorption in the proximal tubule. Am J Physiol Ren Physiol.

[43] Khalil R, Boels MGS, Bezuijen A, Boers JE, de Bruin PC, van Dijk MAAM (2022). Mutations in the heparan sulfate backbone elongating enzymes EXT1 and EXT2 have no major effect on endothelial glycocalyx and the glomerular filtration barrier. Mol Genet Genomics.

[44] Winterborn MH, Bradwell AR, Chesner IM, Jones GT (1987). The origin of proteinuria at high altitude. Postgrad Med J.

[45] Mogensen CE, Vittinghus E, Sølling K (1979). Abnormal albumin excretion after two provocative renal tests in diabetes: physical exercise and lysine injection. Kidney Int.

[46] Goldberg R, Rubinstein AM, Gil N, Hermano E, Li JP, van der Vlag J (2014). Role of heparanase-driven inflammatory cascade in pathogenesis of diabetic nephropathy. Diabetes.

[47] Vinores SA, Gadegbeku C, Campochiaro PA, Green WR (1989). Immunohistochemical localization of blood-retinal barrier breakdown in human diabetics. Am J Pathol.

[48] Vinores SA, Campochiaro PA, Lee A, McGehee R, Gadegbeku C, Green WR (1990). Localization of blood-retinal barrier breakdown in human pathologic specimens by immunohistochemical staining for albumin. Lab Invest.

[49] Murata T, Ishibashi T, Inomata H (1992). Immunohistochemical detection of extravasated fibrinogen (fibrin) in human diabetic retina. Graefes Arch Clin Exp Ophthalmol.

[50] Alsaad KO, Herzenberg AM (2007). Distinguishing diabetic nephropathy from other causes of glomerulosclerosis: an update. J Clin Pathol.

[51] Bruno G, Merletti F, Biggeri A, Bargero G, Ferrero S, Pagano G (2003). Progression to overt nephropathy in type 2 diabetes: the Casale Monferrato Study. Diabetes Care.

[52] Clark SJ, Keenan TDL, Fielder HL, Collinson LJ, Holley RJ, Merry CLR (2011). Mapping the differential distribution of glycosaminoglycans in the adult human retina, choroid, and sclera. Invest Ophthalmol Vis Sci.

[53] Niemelä H, Elima K, Henttinen T, Irjala H, Salmi M, Jalkanen S (2005). Molecular identification of PAL-E, a widely used endothelial-cell marker. Blood.

[54] Witmer AN, van den Born J, Vrensen GFJM, Schlingemann RO (2001). Vascular localization of heparan sulfate proteoglycans in retinas of patients with diabetes mellitus and in VEGF-induced retinopathy using domain-specific antibodies. Curr Eye Res.

[55] Goldberg S, Harvey SJ, Cunningham J, Tryggvason K, Miner JH (2009). Glomerular filtration is normal in the absence of both agrin and perlecan–heparan sulfate from the glomerular basement membrane. Nephrol Dial Transplant.

[56] Tojo A, Kinugasa S (2012). Mechanisms of glomerular albumin filtration and tubular reabsorption. Int J Nephrol.

[57] Roscioni SS, Heerspink HJL, de Zeeuw D (2014). Microalbuminuria: target for renoprotective therapy PRO. Kidney Int.

[58] Touzani F, Geers C, Pozdzik A (2019). Intravitreal injection of Anti-VEGF antibody induces glomerular endothelial cells injury. Case Rep Nephrol.

[59] Hanna RM, Lopez EA, Hasnain H, Selamet U, Wilson J, Youssef PN (2018). Three patients with injection of intravitreal vascular endothelial growth factor inhibitors and subsequent exacerbation of chronic proteinuria and hypertension. Clin Kidney J.

[60] Gil N, Goldberg R, Neuman T, Garsen M, Zcharia E, Rubinstein AM (2012). Heparanase is essential for the development of diabetic nephropathy in mice. Diabetes.

[61] Bernard D, Méhul B, Delattre C, Simonetti L, Thomas-Collignon A, Schmidt R (2001). Purification and characterization of the endoglycosidase heparanase 1 from human plantar stratum corneum: a key enzyme in epidermal physiology?. J Invest Dermatol.

[62] D’Souza SS, Daikoku T, Farach-Carson MC, Carson DD (2007). Heparanase expression and function during early pregnancy in mice. Biol Reprod.

[63] Shu J, Santulli G (2019). Heparanase in health and disease: the neglected housekeeper of the cell?. Atherosclerosis.

[64] Rivara S, Milazzo FM, Giannini G (2016). Heparanase: a rainbow pharmacological target associated to multiple pathologies including rare diseases. Future Med Chem.

[65] Hammond E, Haynes NM, Cullinane C, Brennan TV, Bampton D, Handley P (2018). Immunomodulatory activities of pixatimod: emerging nonclinical and clinical data, and its potential utility in combination with PD-1 inhibitors. J Immunother Cancer.

[66] Tyler PC, Guimond SE, Turnbull JE, Zubkova OV (2015). Single-entity heparan sulfate glycomimetic clusters for therapeutic applications. Angew Chem Int Ed.

[67] Ferro V, Liu L, Johnstone KD, Wimmer N, Karoli T, Handley P (2012). Discovery of PG545: a highly potent and simultaneous inhibitor of angiogenesis, tumor growth, and metastasis. J Med Chem.

[68] Spijkers-Shaw S, Campbell K, Shields NJ, Miller JH, Rendle PM, Jiao W (2022). Synthesis of novel glycolipid mimetics of heparan sulfate and their application in colorectal cancer treatment in a mouse model. Chem Asian J..

[69] Peck T, Davis C, Lenihan-Geels G, Griffiths M, Spijkers-Shaw S, Zubkova OV (2023). The novel HS-mimetic, Tet-29, regulates immune cell trafficking across barriers of the CNS during inflammation. J Neuroinflammation.

[70] Smith RAA, Luo X, Lu X, Tan TC, Le BQ, Zubkova OV (2023). Enhancing BMP-2-mediated osteogenesis with a synthetic heparan sulfate mimetic. Biomater Adv.

