# Correction: Heparanase inhibition as a systemic approach to protect the endothelial glycocalyx and prevent microvascular complications in diabetes

**DOI:** 10.1186/s12933-024-02170-w

**Published:** 2024-02-20

**Authors:** Monica Gamez, Hesham E. Elhegni, Sarah Fawaz, Kwan Ho Ho, Neill W. Campbell, David A. Copland, Karen L. Onions, Matthew J. Butler, Elizabeth J. Wasson, Michael Crompton, Raina D. Ramnath, Yan Qiu, Yu Yamaguchi, Kenton P. Arkill, David O. Bates, Jeremy E. Turnbull, Olga V. Zubkova, Gavin I. Welsh, Denize Atan, Simon C. Satchell, Rebecca R. Foster

**Affiliations:** 1 Bristol Renal, Bristol Medical School, University of Bristol, Dorothy Hodgkin Building, Whitson Street, Bristol, BS1 3NY UK; 2 Department of Computer Science, Merchant Venturers Building, University of Bristol, Woodland Road, Bristol, BS8 1UB UK; 3 Academic Unit of Ophthalmology, Translational Health Sciences, Bristol Medical School, University of Bristol, Biomedical Sciences Building, University Walk, Bristol, BS8 1TD UK; 4 Sanford Burnham Prebys Medical Discovery Institute, 10901 North Torrey Pines Road, CA La Jolla, 92037 USA; 5 School of Medicine, Biodiscovery Institute, University of Nottingham, Nottingham, NG7 2UH UK; 6 Centre for Glycoscience, School of Life Sciences, Keele University, Staffordshire, ST5 5BG UK; 7 Ferrier Research Institute, Victoria University of Wellington, 69 Gracefield Rd, Lower Hutt, 5046 New Zealand; 8 Bristol Eye Hospital, University Hospitals Bristol & Weston NHS Foundation Trust, Bristol, BS1 2LX UK

Following publication of the original article [1], the author noticed the error in Discussion section.

In the last paragraph under Discussion section, the sentence “The heterogenous nature of oligo-/polysaccharide derivatives such as PG545 also results in batch variations, further complicating FDA approval” is incorrect. In this text, PG545 should be replaced with SST0001 and should read “The heterogenous nature of oligo-/polysaccharide derivatives such as SST0001 also results in batch variations, further complicating FDA approval”.
