# Supplementary material for: Heparanase inhibition as a systemic approach to protect the endothelial glycocalyx and prevent microvascular complications in diabetes
Source: Cardiovasc Diabetol. 2024 Feb 1;23:50. doi: 10.1186/s12933-024-02133-1 (PMC10835837; doi:10.1186/s12933-024-02133-1)
Supplement: Supplementary file 1 — Additional file 1: Figure S1. Measurements of glomerular filtration parameters and glycocalyx depth. a Area of measurements (dotted lines) for podocyte foot process (PFP) width, slit diagraph (SD) width, basement membrane (BM) thickness, and length of BM. Number of fenestrations were counted as indicated, and divided by the length of BM to obtain fenestrations/µm. b For eGlx measurements, a grid was overlayed over the image. Red arrows point to examples of eGlx staining. Measurements were taken where the intersecting grid lines intersected the lipid bilayer, examples indicated by (x) and measurement example indicated by dotted lines on inset image. Figure S2. Structure of heparanase inhibitor OVZ/HS-1638. OVZ/HS-1638 is a single entity chemical polyvalent dendrimer which allows for multiple target interactions at multiple points on the inhibitor [29]. Figure S3. LEL stains eGlx components in the retina. Mouse retinal vessel on retinal flat mount stained with FITC-LEL (green) and DAPI (blue). Lumen (Lm) of vessel indicated. Arrow points to eGlx staining on surface of endothelial cell. Figure S4. A vascular bolus of heparinase III has no impact on podocyte glycocalyx or other ultrafiltration parameters. Glomerular filtration barrier measurements in mice treated with inactive or active heparinase III. a Podocyte glycocalyx depth. b Basement membrane (BM) thickness. c Fenestration density. d Slit diaphragm width. e Podocyte foot process width. No statistically significant differences found (n = 5 mice, unpaired t- test, not significant). Figure S5. Ext1(ECKO) mice have no gross changes in retinal or glomerular morphology and have reduced eGlx HS. a Representative optical coherence tomography (OCT) images of retinas from LMC and Ext1(ECKO) mice, showing no changes in morphology. Scale bar 50 µm. b Representative images of cortex Haematoxylin and Eosin staining at × 10 and × 40 magnification are shown for LMC and Ext1(ECKO) mice, showing no change in gross morphology. c R [file 12933_2024_2133_MOESM1_ESM.docx]

**Methods S1**

Processing of samples for transmission electron microscopy

Samples were washed in 0.1M NaCa Buffer and incubated in 1% OsO_4_ in 0.1M NaCa Buffer for 1 hour, washed in NaCa buffer, followed by a de-ionized water wash. Samples were incubated in 3% aqueous uranyl acetate (UA) in the dark for 30 minutes and washed with de-ionized water. Samples were dehydrated though a series of increasing concentration of ethanol washes (70%,80%,90%,96%,100%) followed by Propylene Oxide washes for 10 minutes, three times each. Samples were then incubated overnight in a 1:1 mixture of Epon and Propylene Oxide. Samples were switched over to fresh 100% Epon and incubated overnight. Samples were embedded in fresh Epon and allowed to cure for 72 hours at 60°C. Blocks were trimmed and sectioned at 70nm. Sections were mounted on copper grids and stained in UA followed by washes in de-ionized water. Grids were stained once more with a lead stain composed of 1mL Sodium citrate, 1 mL lead nitrate, and 0.38mL of NaOH followed by washes in de-ionized water. Slides were air dried before imaging.

Electron microscopic glomerular filtration barrier measurements

All images were blinded for analysis and performed using ImageJ image processing program (SciJava software ecosystem, Maryland, USA) as previously described (1). A minimum of three capillaries per glomerulus were measured for each animal and a minimum of five fields of view were analysed per capillary. The number of fenestrations were counted and measurements for basement membrane thickness, slit diaphragm width, and podocyte foot process width measured from relevant lipid bilayer to lipid bilayer as shown in Supplementary Methods Figure 1.

On TEM images, in addition to blinding of images, a grid was overlayed using ImageJ to ensure unbiased measurements of glycocalyx depth. Depth measurements were taken where intersecting grid lines also intersected the lipid bilayer, as shown in (Supplementary Methods Figure 1). Animal glycocalyx depth was calculated by averaging all capillary averages for that animal.


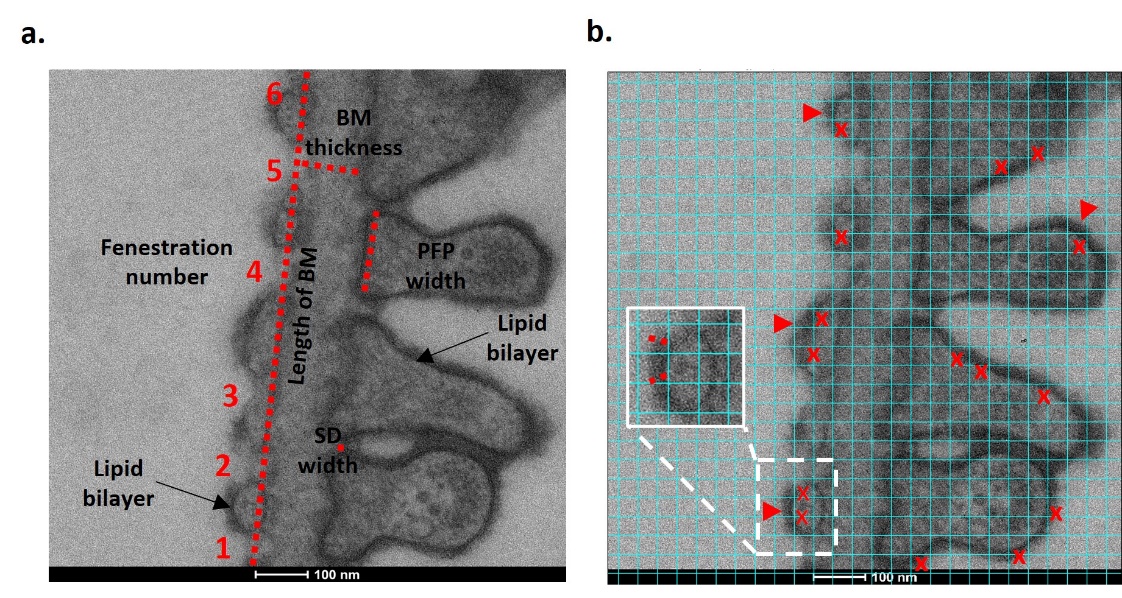


**Figure S1. Measurements of glomerular filtration parameters and glycocalyx depth**(a) Area of measurements (dotted lines) for podocyte foot process (PFP) width, slit diagraph (SD) width, basement membrane (BM) thickness, and length of BM. Number of fenestrations were counted as indicated, and divided by the length of BM to obtain fenestrations/µm. (b) For eGlx measurements, a grid was overlayed over the image. Red arrows point to examples of eGlx staining. Measurements were taken where the intersecting grid lines intersected the lipid bilayer, examples indicated by (x) and measurement example indicated by dotted lines on inset image.

Immunofluorescence analysis of eGlx HS in *Ext1^(ECKO)^* and LMC mice

Kidney tissue from *Ext1^(ECKO)^* and LMC mice were stained with anti-HS3A8V as described in main methods. Confocal images were taken using the Multi-Laser CLSM Leica SP5 (UOBWB) using the 100x oil immersion objective with resolution set to 2048 x 2048. Images were analysed using ImageJ. Frome one kidney, a minimum of three glomeruli were measured per animal. On images of glomeruli stained with R18, a minimum of three capillaries per glomeruli were randomly selected and the luminal area traced around (excluding red blood cells which also contain HS on their surface). Retaining the traced area, the image was then switched to HS staining and area and integrated density (IntDen) were measured. Four background measurements were taken. Corrected total fluorescence (CTF) was calculated by subtracting the IntDen from the area measured multiplied by the mean of fluorescence of background readings.

Isolation of CD31+ glomerular endothelial cells (GEnCs) from *Ext1^(ECKO)^* Mice

Glomeruli were isolated from *Ext1^(ECKO)^* and LMC mouse kidneys using graded sieving and collected from a 75µm pore sieve. GEnCs were fluorescently labelled and sorted by flow cytometry, as previously described (2). Briefly, glomeruli were enzymatically digested with 1mg/ml collagenase (I,V,VI) (Sigma-Aldrich, Dorset, UK) and single cell suspension obtained. Cells were washed and immunostained with PE rat anti-mouse CD31 (BD Pharmingen, San Jose, CA) at 1:50 for 1 hour and washed. A Becton Dickinson Influx Cell Sorter (BD Bioscience, San Jose, CA) was used to isolate CD31 positive cells at The University of Bristol Flow Cytometry Facility.

RNA extraction and real-time quantitative (q) PCR

RNA was extracted from FACS mouse GEnC cells, using RNeasy Mini Kit (Qiagen, Manchester, UK) following manufacturer’s instructions. A total of 2 µg of RNA was converted to cDNA using high-capacity RNA to cDNA conversion kit (Applied Biosystems, Foster City, CA) following manufacturer’s instructions. For qPCR, SYBR Green Master Mix was used (S-4438, Sigma-Aldrich) following manufacturer’s instructions using StepOne 96-well plate real-time PCR system (Life Technologies- Applied Biosystems, Foster City, CA). *Ext1* primer sequences: forward 3’ GTCATCCATGCTGTGACTCC; reverse 3’ GGCTTGTCACAATTCCACAG and *β-Actin* primer sequences: forward 3’CTGTCCCTGTATGCCTCTG; reverse 3’ ATGTCACGCACGATTTCC. Relative fold change (2^-ΔΔCT^) was calculated and *Ext1* values normalised to β-Actin reference gene.

*In vitro* heparanase and OVZ/HS-1638 treatments in GEnC cultures

Human conditionally immortalised GEnC, previously characterized in detail (3), were used for all cell culture studies. Cells were seeded on coverslips and switched from 33°C to 37°C at 70% confluency and left for five days before treatments. Treatments were performed in complete serum free media. Cells were treated with serum free media only (vehicle), or with 500ng/mL active human heparanase (7570-GH, R&D Systems, Abingdon, UK) ± 40µM OVZ/HS-1638 for 1 h at 37°C. Cells were washed with PBS before proceeding to immunofluorescence.

*In vitro* GEnC HS immunofluorescence and analysis

Cells were fixed with 4% PFA and stained with the anti-HS antibody 10E4 at 1:100 (H1890-USB, Stratech Scientific Ltd, Ely, UK). Secondary anti-mouse488 (A28175, ThermoFisher Scientific) was incubated at 1:250. Coverslips were counterstained with DAPI (ThermoFisher Scientific) and mounted with Pro long gold (P10144, ThermoFisher Scientific). Images were taken with 63X objective on a Multi-Laser CLSM Leica SP5 confocal microscope (Leica Microsystems, Wetzlar, Germany). A minimum of three fields of view were taken per coverslip. For analysis, surface HS signal was measured using ImageJ and total CTF calculated as above, and normalised to nuclei number.

Periodic acid Schiff’s (PAS) staining

Paraffin embedded mouse kidney sections were deparaffinized in Xylenes and rehydrated through a series of decreasing ethanol concentrations. Histological PAS staining was carried out and glomerular PAS staining was quantified as previously (4). A minimum of three glomeruli were analysed per mouse.

**Figures**

**
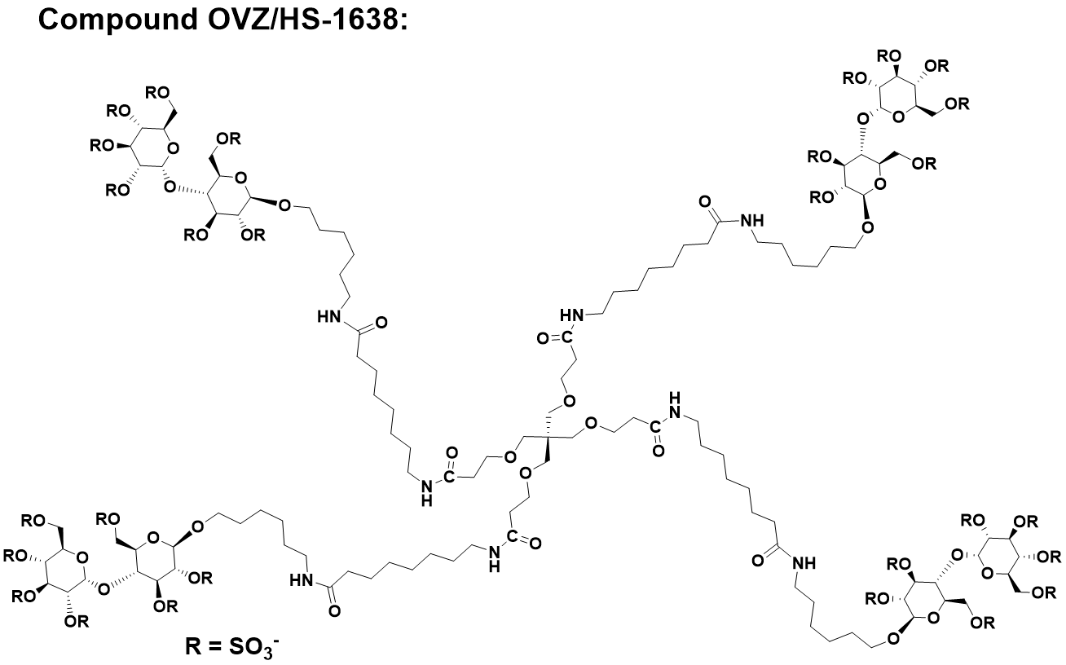
**

**Figure S2.** **Structure of heparanase inhibitor OVZ/HS-1638**. OVZ/HS-1638 is a single entity chemical polyvalent dendrimer which allows for multiple target interactions at multiple points on the inhibitor (29).


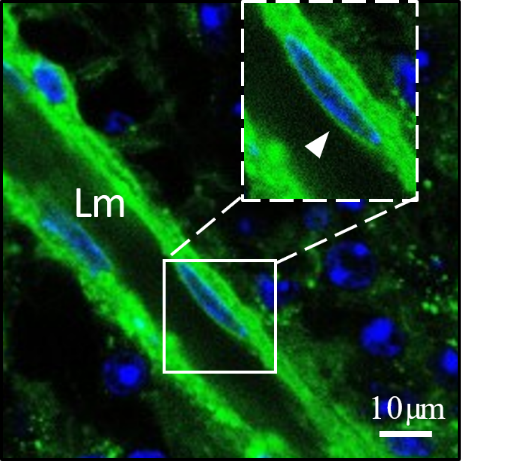


**Figure S3. LEL stains eGlx components in the retina**Mouse retinal vessel on retinal flat mount stained with FITC-LEL (green) and DAPI (blue). Lumen (Lm) of vessel indicated. Arrow points to eGlx staining on surface of endothelial cell.

**Figure S4.** **A vascular bolus of heparinase III has no impact on podocyte glycocalyx or other ultrafiltration parameters**. Glomerular filtration barrier measurements in mice treated with inactive or active heparinase III. (a) Podocyte glycocalyx depth (b) Basement membrane (BM) thickness. (c) Fenestration density. (d) Slit diaphragm width. (e) Podocyte foot process width. No statistically significant differences found (n=5 mice, unpaired t- test, not significant).


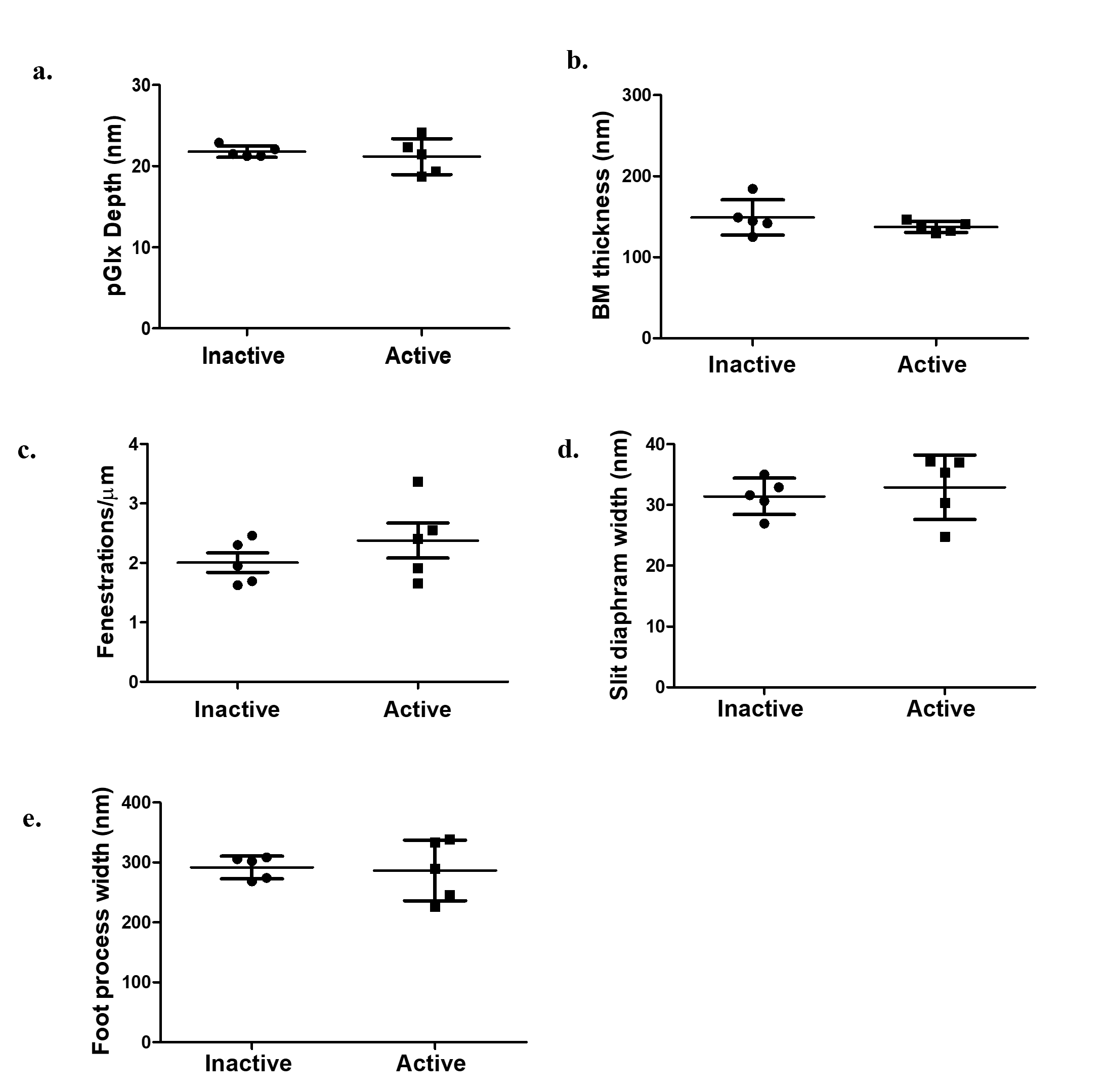


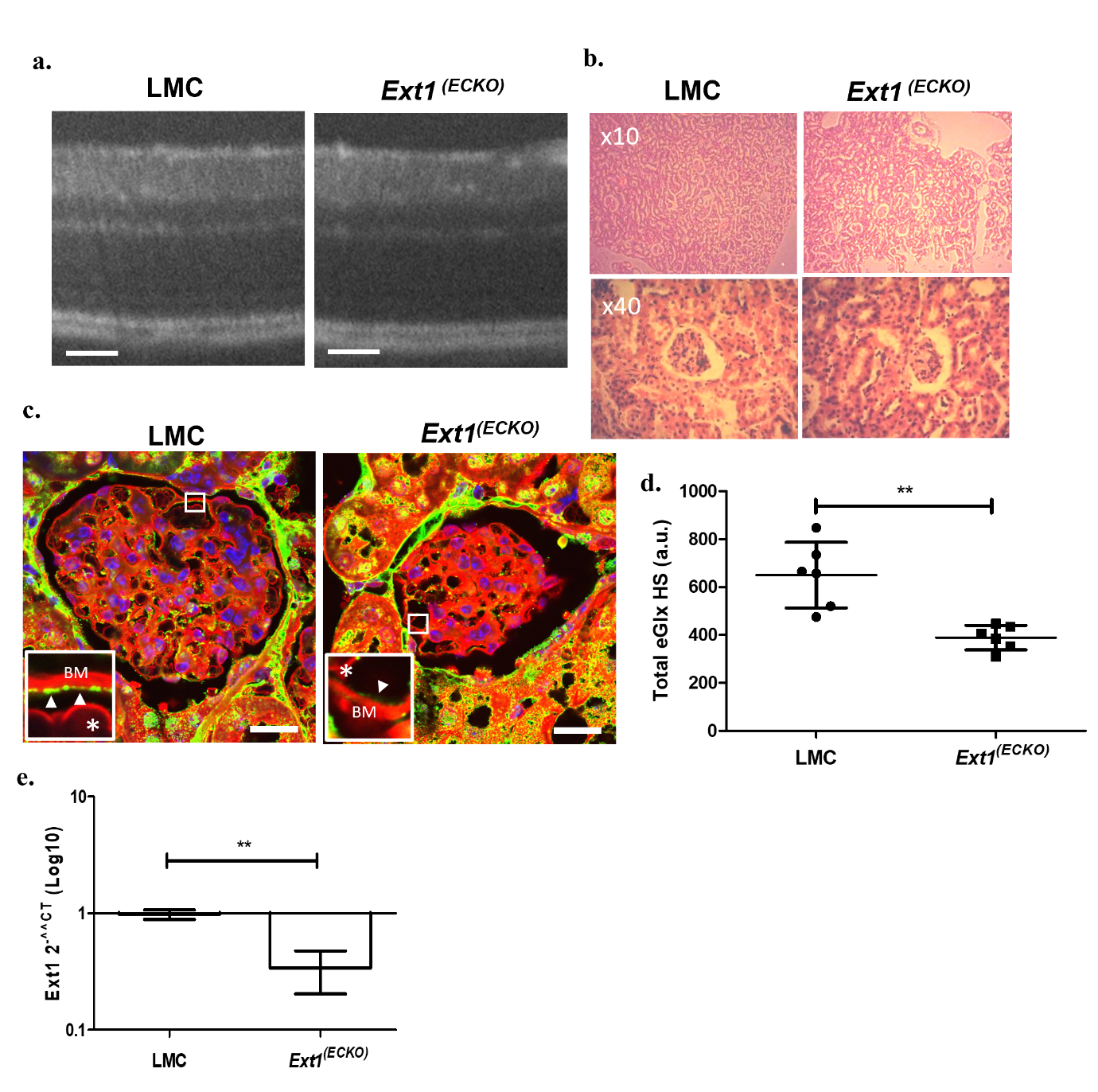


**Figure S5. *Ext1^(ECKO)^*** **mice have no gross changes in retinal or glomerular morphology and have reduced eGlx HS.** (a) Representative optical coherence tomography (OCT) images of retinas from LMC and *Ext1^(ECKO)^* mice, showing no changes in morphology. Scale bar 50µm. (b)Representative images of cortex Haematoxylin and Eosin staining at x10 and x40 magnification are shown for LMC and *Ext1^(ECKO)^* mice, showing no change in gross morphology. (c) Representative images of heparan sulphate staining on kidney from LMC and *Ext1^(ECKO)^* mice. Inset shows eGlx heparan sulphate staining (green) on luminal side of capillary (arrowhead). Membrane stain R18 (red) used to stain cell membranes and DAPI for nuclear staining (blue). Red blood cells (*) and basement membrane (BM) indicated. Scale bar = 20µm (d) Analysis of glomerular capillary eGlx heparan sulphate staining. (n=6 mice, **p<0.01, unpaired t-test). (e) Quantitative PCR performed on FAC sorted glomerular endothelial cells from littermate control (LMC) and *Ext1^(ECKO)^* mice. (n=3 mice, **p<0.01, unpaired t-test).

**Figure S6. *Ext1^(ECKO)^* mice have no change in podocyte glycocalyx or other ultrafiltration parameters.** Glomerular filtration barrier measurements in littermate control (LMC) and *Ext1^(ECKO)^* mice. (a) Podocyte glycocalyx depth. (b) Basement membrane (BM) thickness. (c) Fenestration density. (d) Slit diaphragm width. (e) Podocyte foot process width (n=6 mice/group, unpaired t- test, not significant).


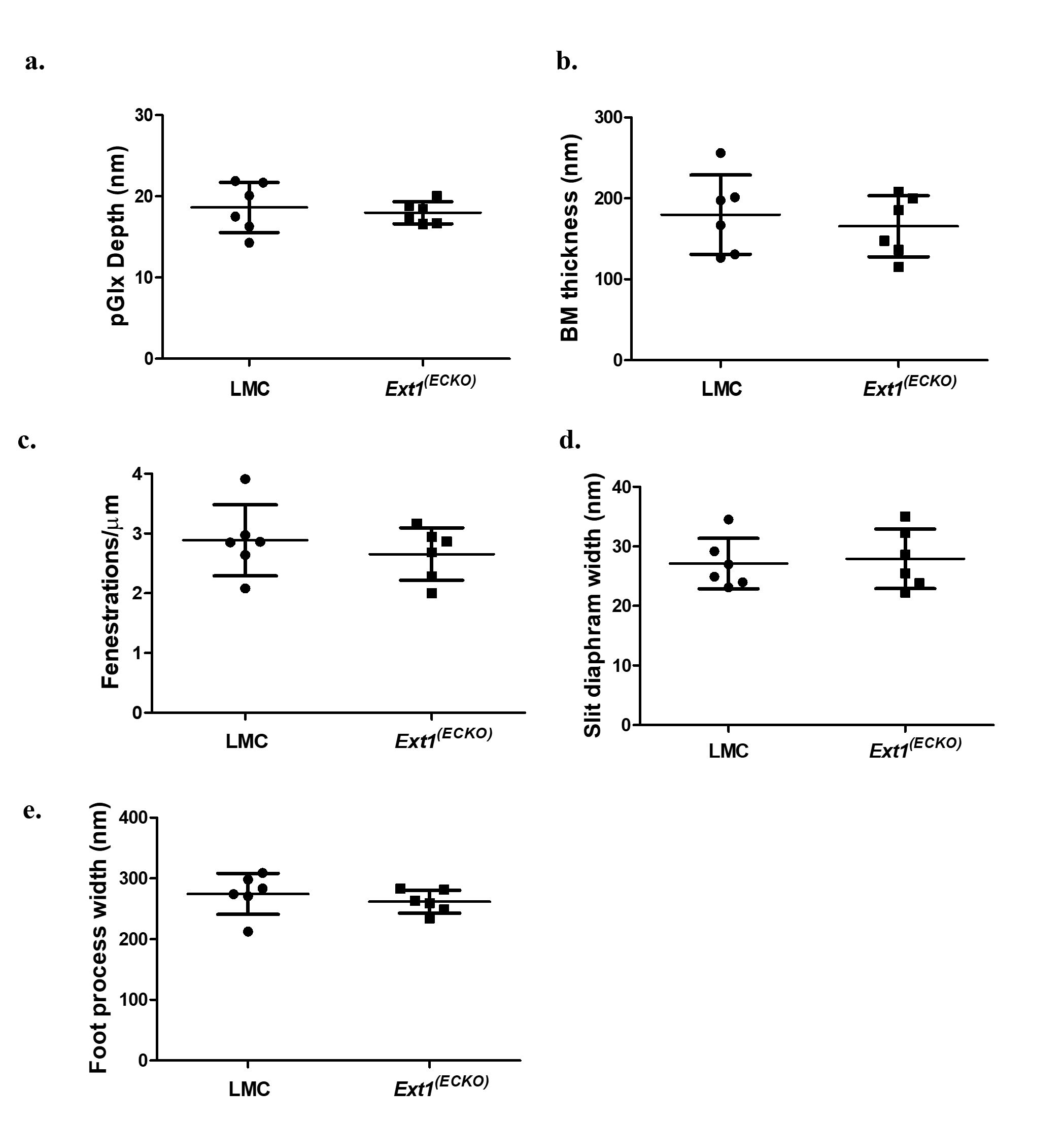

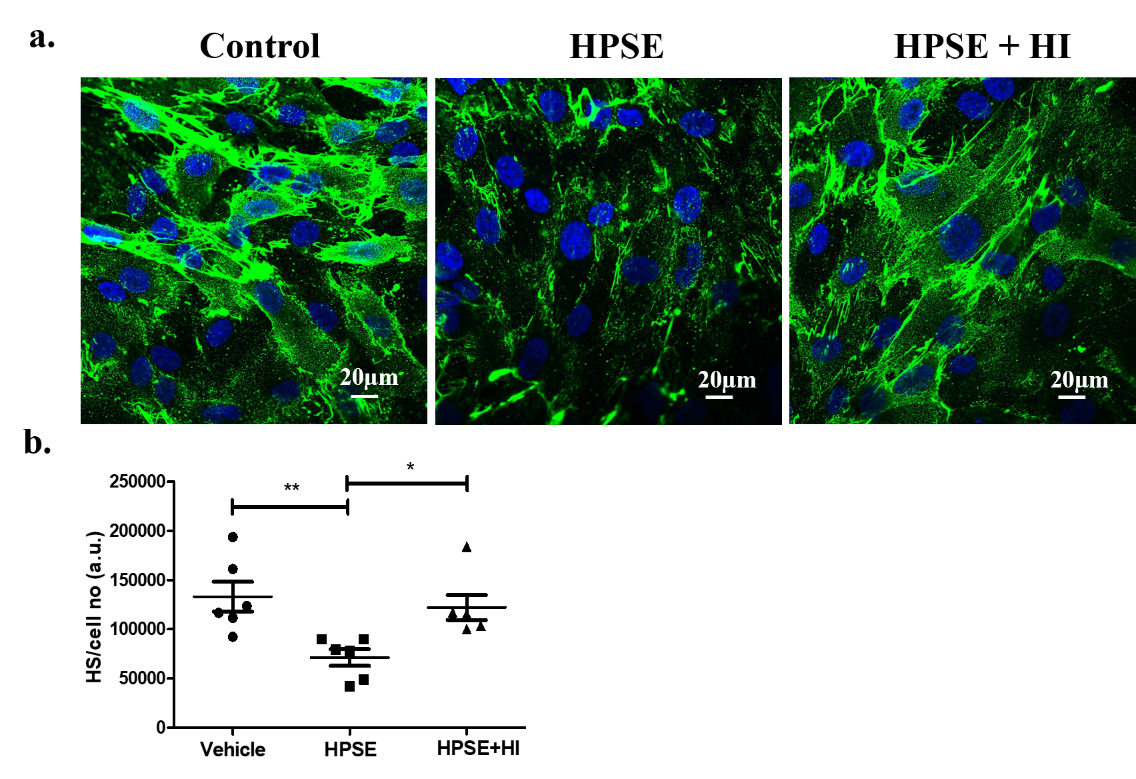


**Figure S7. Treatment with the heparanase inhibitor OVZ/HS-1638 *in vitro* prevents heparan sulphate shedding by heparanase.** (a) Representative confocal images of control, heparanase (HPSE), or HPSE + OVZ/HS-1638 inhibitor (HPSE + HI) treated GEnCs stained with anti-heparan sulphate in green. (b) Quantification of total heparan sulphate staining in Control, HPSE, and HPSE + HI treated GEnCs, normalized to cell number (n=6 technical repeats, *P*<*0.05,***P<*0.01, One way ANOVA for normally distributed data, Tukey’s multiple comparison test).


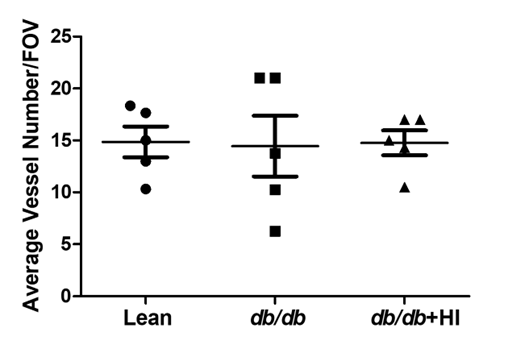


**Figure S8. Vessel number is not significantly impacted in *db/db* mice at 11-weeks-of-age** Average number of vessels counted per animal in albumin-stained retina per field of view (FOV), minimum of three FOV counted per animal (n=6 mice/group, One-way ANOVA with Tukey’s multiple comparison test. Not significant).

**Figure S9.** **Db/db mice develop early signs of diabetic nephropathy.**

Glomerular filtration barrier measurements for lean, diabetic (*db/db*) and diabetic mice treated with OVZ/HS-1638 (*db/db* + HI). (a) Podocyte glycocalyx depth. (b) Basement membrane (BM) thickness. (c) Fenestration density (d) Slit diaphragm width (e) Podocyte foot process width (n=5 mice/group. One way ANOVA for normally distributed data, Tukey’s multiple comparison test. No statistically significant differences found for a-b. For d,e, * *P*<0.05, ** *P*< 0.01).


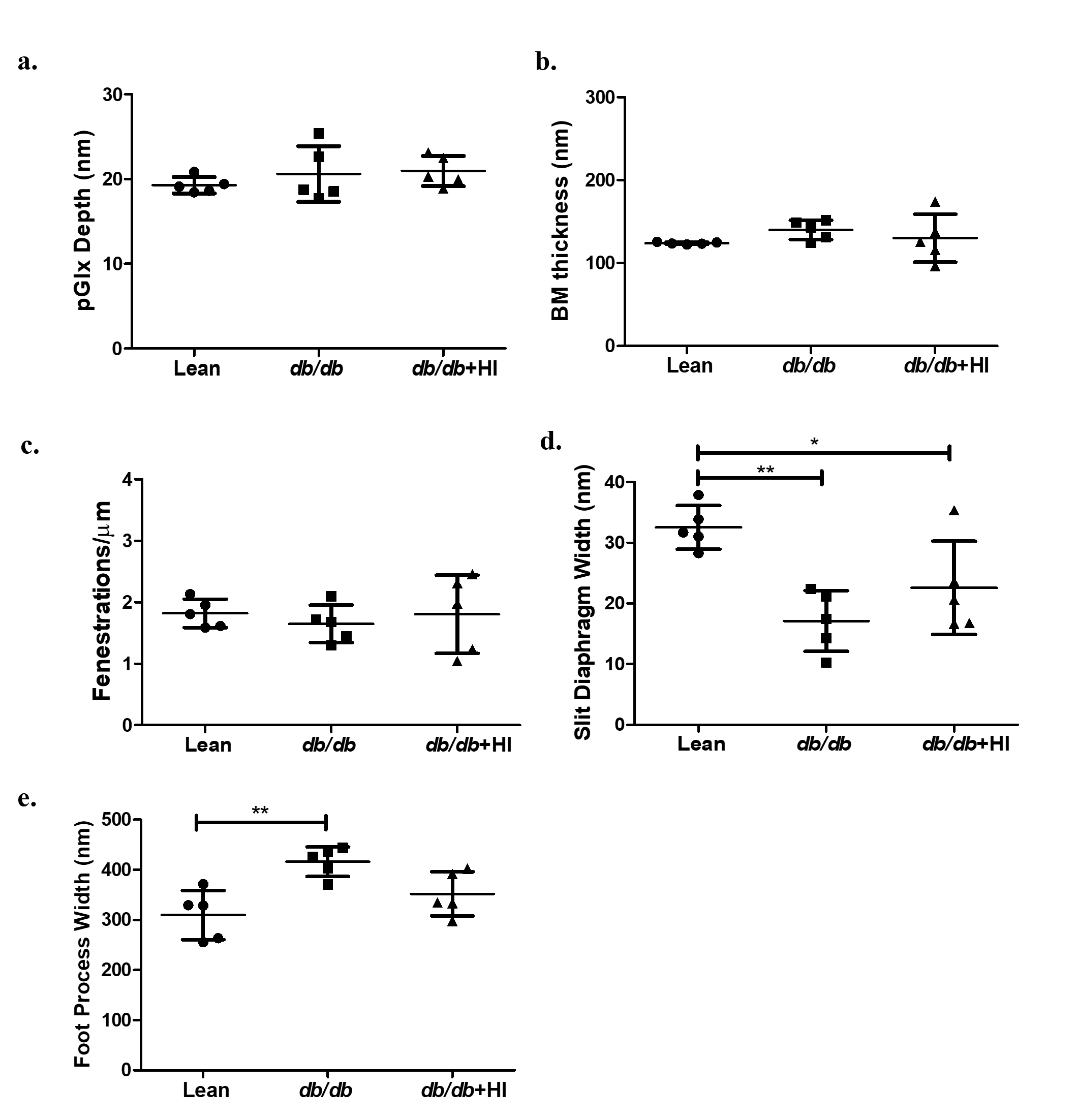

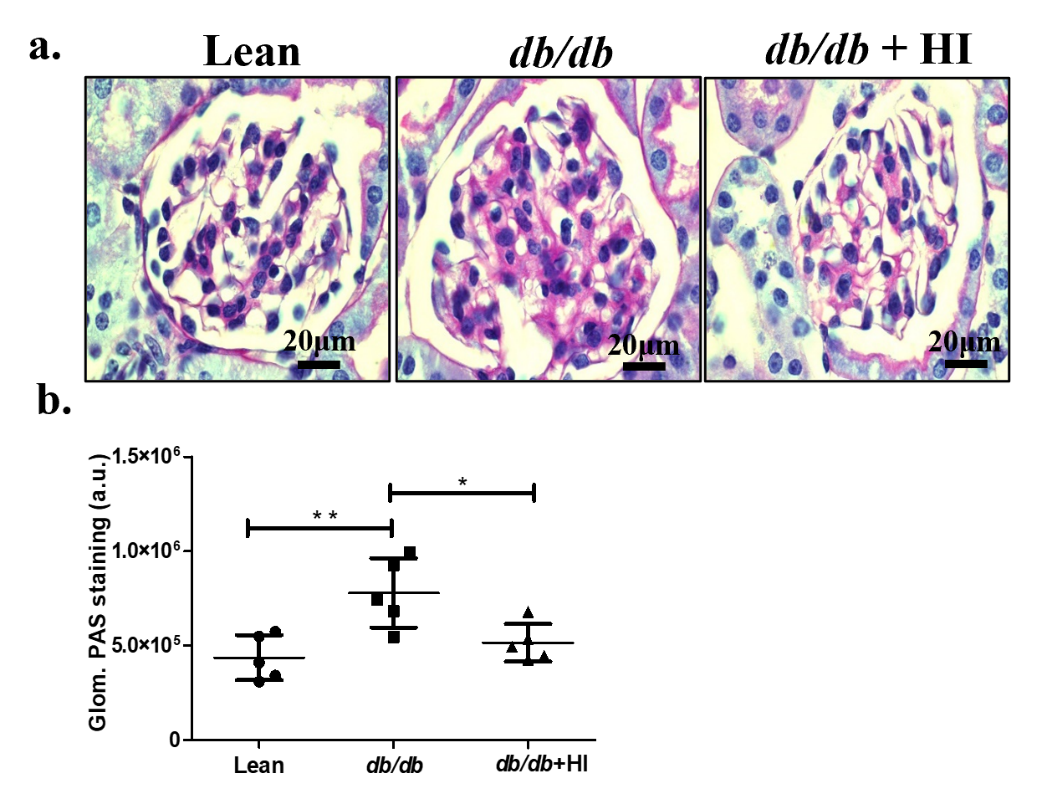


**Figure S10. Treatment with OVZ/HS-1638 prevents glycogen deposition in diabetic nephropathy in *db/db* mice.** (a) Representative Periodic acid-Schiff (PAS) staining images for lean, diabetic (*db/db*) and diabetic treated with OVZ/HS-1638 (*db/db* + HI) (b) PAS staining analysed to measure fibrosis within glomerulus in lean, diabetic (*db/db*) and diabetic treated with OVZ/HS-1638 (*db/db* + HI) mice. (n=5 mice/ group, **P*<0.05 ***P*<0.01, One way ANOVA for normally distributed data, Tukey’s multiple comparison test).

References

1. Oltean S, Qiu Y, Ferguson JK, Stevens M, Neal C, Russell A, et al. Vascular Endothelial Growth Factor-A165b Is Protective and Restores Endothelial Glycocalyx in Diabetic Nephropathy. J Am Soc Nephrol. 2015 Aug;26(8):1889–904.

2. Ramnath RD, Butler MJ, Newman G, Desideri S, Russell A, Lay AC, et al. Blocking matrix metalloproteinase-mediated syndecan-4 shedding restores the endothelial glycocalyx and glomerular filtration barrier function in early diabetic kidney disease. Kidney Int. 2020 May;97(5):951–65.

3. Satchell SC, Tasman CH, Singh A, Ni L, Geelen J, von Ruhland CJ, et al. Conditionally immortalized human glomerular endothelial cells expressing fenestrations in response to VEGF. Kidney International. 2006 May 1;69(9):1633–40.

4. Onions KL, Gamez M, Buckner NR, Baker SL, Betteridge KB, Desideri S, et al. VEGFC Reduces Glomerular Albumin Permeability and Protects Against Alterations in VEGF Receptor Expression in Diabetic Nephropathy. Diabetes. 2019 Jan 1;68(1):172–87.
